# Efficient and Reusable Magnetic Sulfonated Biochar from Amazonian Waste for Sustainable Production of Glycerol Acetals: Statistical Optimization of Catalytic Process

**DOI:** 10.1021/acsomega.6c06154

**Published:** 2026-09-17

**Authors:** Thaissa Saraiva Ribeiro, Izadora de Araújo Sobrinho, Ane Caroline Dias e Silva, Beatriz dos Santos Silva, Deborah da Cunha Fonseca, Vicente da Silva Lima, Matheus Arrais Gonçalves, Alcineia Conceição Oliveira, Igor Alexandre Rocha Barreto, Marcondes Lima da Costa, Leyvison Rafael Vieira da Conceição

**Affiliations:** † Federal University of Pará, Institute of Exact and Natural Sciences, Graduate in Chemistry Program, Laboratory of Catalysis and Oleochemical, 66075−110 Belém, Pará, Brazil; ‡ Federal Institute of Education, Science and Technology of Pará, Graduate in Materials Engineering Program, 66093−020 Belém, Pará, Brazil; § Federal University of Pará, Institute of Biological Sciences, Graduate Program in Biotechnology, Amazon Oils Laboratory, 66075−110 Belém, Pará, Brazil; ∥ Federal University of Ceará, Science Center, Department of Analytical Chemistry and Physical Chemistry, Graduate Program in Chemistry, Physical Chemistry Laboratory, 60440-900 Fortaleza, Ceará, Brazil; ⊥ Federal University of Ceará, Science Center, Department of Analytical Chemistry and Physical Chemistry, Graduate Program in Chemical Engineering, Physical Chemistry Laboratory, 60440-900 Fortaleza, Ceará, Brazil; # Federal University of Pará, Program for Post-graduation in Geology and Geochemistry, Institute of Geosciences, 66075-110, Belém, Pará, Brazil

## Abstract

Glycerol valorization, the main coproduct of biodiesel production, has driven the development of catalytic routes to obtain higher value-added products, such as acetals. This study reports the synthesis of a novel magnetic heterogeneous acid catalyst derived from a sulfonated biochar obtained from an Amazonian agro-industrial waste and impregnated with nickel ferrite (NiFe_2_O_4_). Characterization techniques (surface acidity, XRD, FT-IR, SEM/EDS, and VSM) confirmed the effective sulfonation and magnetization of the CM750 biochar. Optimization of glycerol acetylation with acetic acid was performed using Taguchi orthogonal design. Under optimized conditions (120 °C, 2.5 h, 7.5% catalyst, 1:5 glycerol:acetic acid molar ratio), the CMS750-NiFe_2_O_4_ catalyst achieved 99.79% glycerol conversion, with high selectivity for DAG (40.16%) and TAG (50.16%) and low selectivity for MAG (9.58%). The predictive models developed for glycerol conversion and MAG, DAG, and TAG selectivities exhibited high goodness of fit (adjusted *R* ^2^ > 0.95) and relative errors below 5%. The CMS750-NiFe_2_O_4_ catalyst maintained catalytic activity after four reuse cycles, with glycerol conversion above 81% and selectivities of 65.44% (MAG), 35.82% (DAG), and 1.75% (TAG). Thus, Amazonian agro-industrial waste proves viable as a renewable source for developing efficient, sustainable, and low-cost catalysts.

## Graphical abstract

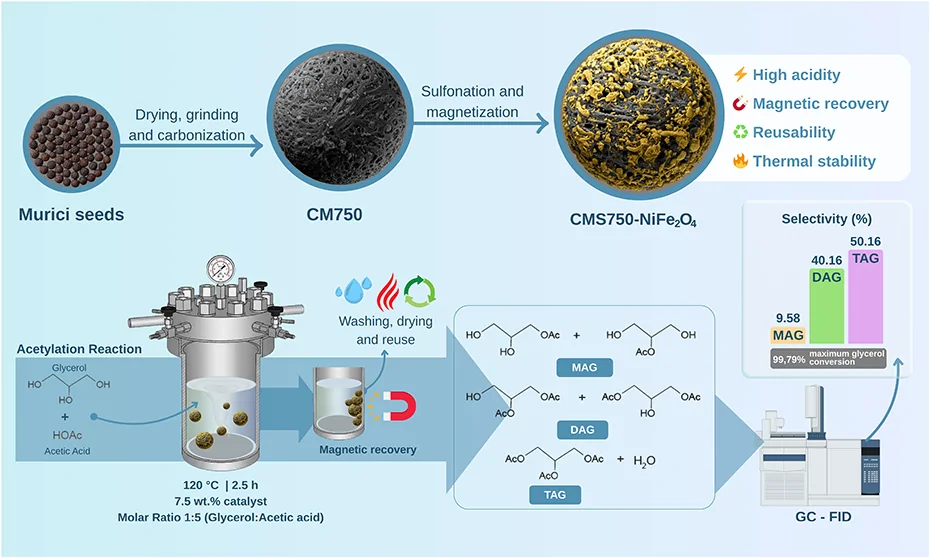

## 1 Introduction

Population growth and increasing urbanization worldwide have stimulated the demand for alternative energy sources, particularly for the replacement of fossil fuels.^1^ In this scenario, biodiesel has been establishing itself in the fuel market as an alternative to fossil diesel since its combustion emits lower amounts of greenhouse gases, such as carbon monoxide, carbon dioxide (CO_2_), and nitrogen dioxide (NO_2_).^2^ Furthermore, it possesses physicochemical and fuel properties similar to those of conventional diesel, thus being suitable for use in combustion engines either blended with fossil diesel or in its pure form, without the need for mechanical modifications to these engines.^3^

The production of biodiesel via the transesterification reaction has become industrially well-established; however, this process generates a large volume of glycerol, its main coproduct.^4^ During the process, approximately 10% of crude glycerol is generated per ton of biodiesel produced. Consequently, biodiesel-producing industries face a major challenge regarding the management of the surplus of this coproduct.^5,6^ In this context, the wide availability and low cost make glycerol an economically viable feedstock for application in various chemical processes aimed at its conversion into high-value commercial products.^7^

Among the main chemical processes for the catalytic conversion of glycerol, the acetylation reaction stands out for promoting the efficient conversion of glycerol to products of greater industrial interest, such as monoacetin, diacetin, and triacetin.^8^ The isomers 1-monoacetin and 2-monoacetin constitute raw materials used in the pharmaceutical industry, in the manufacture of explosives, and as food additives.^10,11^ The isomers 1,2-diacetin and 1,3-diacetin are widely employed as plasticizing coatings, food additives, or intermediates in the production of structural lipids and cosmetics.^9,12^ Triacetin is used as a fuel additive to increase the octane number of gasoline and as a gelling agent.^13,14^ Finally, diacetin and triacetin offer an alternative to the use of tertiary alkyl ethers by improving cold flow properties, reducing viscosity, and increasing octane rating while also contributing to the reduction of particulate matter, hydrocarbon, and carbon monoxide emissions.^15,16^

Acetylation is an esterification reaction in which an acid (such as acetic acid) reacts with an alcohol (glycerol) in the presence of an acid catalyst, resulting in the formation of acetals (monoacetin, diacetin, and triacetin) and water.^17^ Traditionally, this reaction is catalyzed by homogeneous acid catalysts, such as sulfuric acid and p-toluenesulfonic acid, which provide a high process efficiency.^18^ However, they have major disadvantages, including difficulty in separation and recovery, which leads to increased costs in the treatment of the acetals produced.^19^ In this context, the use of heterogeneous acid catalysts emerges as an alternative, as they can be easily recovered and reused, contributing to the reduction of product purification costs, in addition to being more environmentally friendly and economical.^20^

Numerous heterogeneous acid catalysts have been applied in the glycerol acetylation reaction, including heteropolyacids,^21^ metal oxides,^6,15^ graphene-based materials,^18^ sulfonated resins,^10^ and sulfonated carbons.^4,17^ Among the employed materials, sulfonated carbons derived from residual biomass stand out, since the sulfonic groups anchored to the carbonaceous matrix impart strong acidity, high thermal and chemical stability, as well as lower leaching tendency when compared to other heterogeneous catalysts.^21,22^ Furthermore, the incorporation of a magnetic phase into these sulfonated catalysts provides the advantage of separation through the application of an external magnetic field, eliminating the need for conventional recovery routes (such as filtration or centrifugation), facilitating the recovery and reuse processes of the solids, reducing solvent consumption and purification costs, and making the process economically and environmentally more favorable.^23,24^

The murici tree (*Byrsonima crassifolia*) is a small-sized tree species native to South America and widely distributed in the Amazon region.^3^ This tree can reach 2–6 m in height, and its fruits are rounded, measuring 1.5–2 cm in diameter, and when ripe, they exhibit a yellowish color with a characteristic aroma and flavor.^25^ Fruiting of the murici tree occurs from November to May, with an average production of 12 kg per tree, and murici fruits are widely appreciated fresh or in processed products, such as juices, jams, ice creams, and liqueurs.^27^ The pulp corresponds to approximately 70.9% of the fruit, while the seed represents 29.1%, characterizing it as an agro-industrial residue with high potential for the synthesis of heterogeneous catalysts, still underexplored in the literature.^26^

In this context, this study aims to synthesize a novel sulfonated acid catalyst based on biochar derived from murici seeds and apply it to the esterification reaction of glycerol with acetic acid for the production of acetals. Finally, it seeks to evaluate and optimize the reaction parameters using the Taguchi L9 optimization method as well as to study its reusability in acetal production.

## 2 Materials and Methods

### 2.1 Materials

Murici fruits were obtained from a local market in the city of Belém (Pará, Brazil). For the sulfonation process, concentrated sulfuric acid (H_2_SO_4_, ÊXODO CIENTIFICA, 98.0%) was used. For the synthesis of the magnetic phase, iron­(III) chloride (FeCl_3_, Sigma-Aldrich, 98.0%), and nickel nitrate hexahydrate (Ni­(NO_3_)_2_·6H_2_O, Merck, 99.8%) were employed. For the acetylation reactions, glacial acetic acid (ÊXODO CIENTIFICA) and glycerol (ACS Científica, 99.5%) were used. For the catalyst recovery process, ethyl alcohol (C_2_H_5_OH, Dinâmica, 99.8%) was employed. Hydrochloric acid (HCl, Qhemis, 37.0%) and sodium hydroxide (NaOH, Neon, 98.0%) were used for the determination of surface acidity of the synthesized materials.

### 2.2 Synthesis of the CMS750-NiFe_2_O_4_ Catalyst

#### 2.2.1 Synthesis of CM750 Biochar

The carbonization process was carried out according to the methodology described by Ribeiro et al.,^27^ with minor adaptations. Initially, the fruits were pulped, and the seeds were washed under running water to remove dirt and pulp residues. Subsequently, the biomass was dried in an oven at 80 °C for 24 h. After this step, the dried seeds were ground in an analytical mill and then sieved through a 10 mesh. The biomass was then carbonized in a tubular furnace at 750 °C for 1 h (heating rate of 10 °C min^–1^) under a nitrogen atmosphere. Finally, the obtained CM750 biochar was cooled to room temperature, placed in a hermetically sealed flask, and stored in a desiccator until use in the catalyst synthesis. Figure 1(a) illustrates the procedure described.

**Figure 1:**
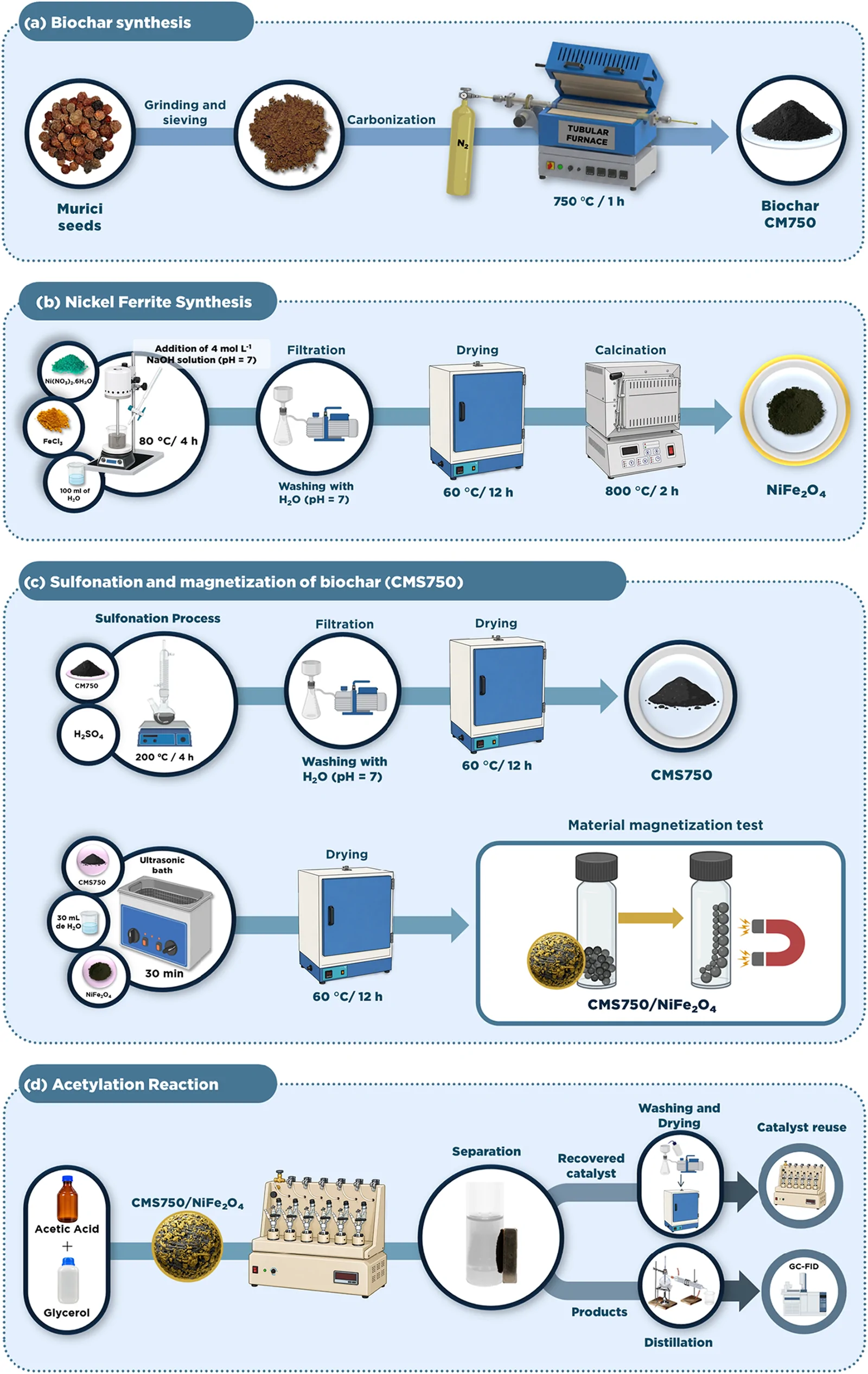
Schematic diagram of the synthesis of (a) biochar CM750, (b) NiFe_2_O_4_, (c) catalyst CMS750-NiFe_2_O_4_, and (d) acetylation reaction.

#### 2.2.2 Synthesis of Nickel Ferrite (NiFe_2_O_4_)

Nickel ferrite was prepared via the coprecipitation method according to the methodology proposed by Gonçalves et al.,^24^ with minor modifications, as shown in Figure 1(b). In a typical synthesis procedure, using an iron:nickel molar ratio of 2:1, a mass of 1.45 g of iron­(III) chloride (FeCl_3_) was dissolved in a beaker containing 100 mL of distilled water under mechanical stirring, followed by the addition of 1.62 g of nickel nitrate hexahydrate (Ni­(NO_3_)_2_·6H_2_O). Subsequently, 10 mL of HNO_3_ was added to reduce the solution pH to 3. Then, a NaOH solution (4 mol L^–1^) was slowly added until the mixture in the beaker reached pH = 12. Immediately after pH stabilization, the mixture was maintained under mechanical stirring at 80 °C for 4 h. After this period, the formed precipitate was filtered, washed with distilled water until pH = 7, dried in an oven at 105 °C for 12 h, and calcined in a muffle furnace at 800 °C for 2 h (heating rate of 10 °C min^–1^). Finally, the nickel ferrite (NiFe_2_O_4_) was cooled, placed in a hermetically sealed flask, and stored in a desiccator until use in the catalyst magnetization process.

#### 2.2.3 Sulfonation and Incorporation of the Magnetic Phase

The synthesis of the CMS750-NiFe_2_O_4_ catalyst (Figure 1(c)) was divided into two steps: the first consisted of the sulfonation of the CM750 biochar, while the second consisted of the incorporation of the magnetic phase (NiFe_2_O_4_) via wet impregnation. The sulfonation process followed the methodology of Corrêa et al.,^28^ using a solid-to-concentrated sulfuric acid ratio of 1:10. In a typical procedure, 1 g of CM750 biochar was dispersed in a flat-bottom flask containing 10 mL of sulfuric acid. The flask was then attached to a reflux system and heated to 200 °C for 4 h under constant stirring. At the end, the obtained material was washed with distilled water until neutral pH and dried at 60 °C for 12 h. The incorporation of the magnetic phase was carried out according to a methodology adapted from Ribeiro et al.^3^ For this purpose, based on 1 g of CM750 biochar used in the sulfonation process, 10% NiFe_2_O_4_ relative to this mass was added to 50 mL of distilled water. The mixture was then kept in an ultrasonic bath at 25 °C for 3 h. Finally, the material was dried in an oven at 60 °C for 24 h. The obtained material was denoted as magnetic sulfonated biochar (CMS750-NiFe_2_O_4_).

### 2.3 Characterization of the Materials

The synthesized materials were characterized by surface acidity (SA) analysis, performed by acid–base titration according to the methodology of Boehm^29^ with adaptations. This method consists of placing a mass of 0.1 g of the sample in a standard NaOH solution (0.1 mol L^–1^), maintaining the system under constant stirring at 25 °C for 24 h. Subsequently, an aliquot of the supernatant is withdrawn and titrated with a standard HCl solution (0.1 mol L^–1^), using phenolphthalein as an indicator. Thermogravimetric Analysis (TGA) was performed on a NETZSCH apparatus, model STA 449F5, in the temperature range from 25 °C to 1000 °C (heating rate of 10 °C min^–1^), with a N_2_ flow of 50 mL min^–1^ and using an alumina crucible. X-ray diffraction (XRD) patterns were obtained using the powder method on a PANalytical diffractometer, model EMPYREAN, with Cu Kα radiation (λ = 1.541874 Å) at 40 kV and 30 mA, and a scanning range of 10° < 2θ < 65°. Fourier-Transform Infrared Spectroscopy (FT-IR) analysis was performed on a BRUKER spectrometer, model VERTEX 70v, using ATR. Spectra were collected in the spectral range of 3000–500 cm^–1^ with a resolution of 4 cm^–1^ and a total of 32 scans. Surface micrographs were obtained by Scanning Electron Microscopy (SEM) using a Tescan microscope, model VEGA 3 LMU, operating with an acceleration voltage of 15 kV. Surface elemental composition was determined by Energy-Dispersive X-ray Spectroscopy (EDS) using an Oxford microanalysis system, model AZTec Energy X-Act, with a resolution of 129 eV. Magnetic properties were determined by Vibrating Sample Magnetometry (VSM) at 25 °C under an applied magnetic field ranging from −20,000 to 20,000 Oersted (Oe), using a MicroSense magnetometer, model EZ9. N_2_ adsorption-desorption isotherms were obtained at 77.3 K using a Micromeritics TriStar II surface area analyzer. Prior to each measurement, the samples were degassed at 200 °C for 2 h to remove physically adsorbed species. The specific surface area was determined using the Brunauer–Emmett–Teller (BET) method, while the pore volume and average pore diameter were calculated using the Barrett–Joyner–Halenda (BJH) method.

### 2.4 Acetylation Reaction

The synthesized materials (CM750 biochar, NiFe_2_O_4_, and CMS750-NiFe_2_O_4_ catalyst) were tested in glycerol acetylation reactions employing acetic acid. The reactions were carried out in batch mode using a PARR Series 5000 Multiple Reactor System, operating at a fixed stirring speed of 900 rpm. Additionally, the influence of reaction variables (reaction temperature, reaction time, catalyst concentration, and glycerol:acetic acid molar ratio) on glycerol conversion and acetal selectivity was evaluated. At the end of each reaction, the catalyst was separated from the reaction products by applying a magnetic field, then washed with portions of distilled water and ethyl alcohol, dried in an oven at 60 °C for 12 h, and reused in a subsequent reaction cycle. Meanwhile, the reaction products were subjected to a simple distillation process to remove residual acetic acid. Finally, the obtained products were analyzed by gas chromatography (GC-FID) to determine glycerol conversion and catalyst selectivity in acetal production. Figure 1(d) illustrates the described procedure.

### 2.5 Determination of Conversion and Selectivity

The determination of glycerol conversion into products was performed by gas chromatography according to the methodology described by Aghbashlo et al.,^30^ with minor adaptations. A Varian CP 3800 gas chromatograph equipped with a flame ionization detector (FID) and a CP WAX 52 CB capillary column (30 m length, 0.32 mm diameter, 0.25 μm film thickness) was used. The oven temperature program started at 80 °C, with a heating rate of 15 °C min^–1^ up to 260 °C (the same temperature as the FID and injector). Helium was used as the carrier gas at a flow rate of 1.3 mL min^–1^, methanol as the solvent, and the injection volume was 1.0 μL.

The reference solution was prepared by dissolving 0.0200 g of glycerol in 8.0 mL of methanol. Subsequently, the resulting solution was transferred to a vial and injected into the chromatograph to obtain the area corresponding to the reference glycerol peak, required for conversion calculation. Then, an aliquot of the sample obtained after the reaction was prepared in the same manner, and the solution was injected into the chromatograph under the same analytical conditions. After the analyses, the glycerol peak was identified in both chromatograms, and the respective peak areas were determined. Glycerol conversion was then calculated based on the ratio between the glycerol peak area of the reaction sample (final area) and the peak area of the reference solution (initial area), as established in eq 1.

1 $$g\mathrm{lycerol}\;\mathrm{conversion}\;\left(\%\right)=\left(-\frac{{AG}_{f}}{{AG}_{i}}\times 100\right)$$

where

AG_*f*_ is the final glycerol peak area (mg L^–1^);

AG_*i*_ is the initial glycerol peak area (mg L^–1^).

Product selectivity was determined based on the chromatographic peak areas corresponding to the formed acetins, monoacetin (MAG), diacetin (DAG), and triacetin (TAG), as identified in the sample chromatogram. The selectivity of each product was calculated individually using eq 2

2 $$selectivity\;\left(\%\right)=\frac{\left[\mathrm{MA}G\right]or\left[DAG\right]\mathrm{or}\left[TAG\right]}{\left[MAG\right]+\left[DAG\right]+\left[TAG\right]}\times 100$$

where [MAG], [DAG], and [TAG] represent the peak areas corresponding to monoacetin, diacetin, and triacetin, respectively, in the reaction product.

### 2.6 Taguchi Orthogonal Design

Among the numerous methodologies applied to process optimization, such as the one-variable-at-a-time (OVAT) method, response surface methodology (RSM), and Box-Behnken (BBD) and central composite (CCD) designs, the Taguchi orthogonal design stands out for its robustness and use of orthogonal arrays.^23,31^ Orthogonal arrays assist in determining the minimum number of experimental trials required for optimizing process parameters without compromising the quality of the obtained response.^32^

Thus, the Taguchi orthogonal design was employed to evaluate the effect of the variables, reaction temperature, reaction time, glycerol:acetic acid molar ratio, and catalyst concentration, on glycerol conversion and acetal formation selectivity. Each independent variable was evaluated at three levels, adopting the following coding and respective ranges: reaction temperature (A: 60–120 °C), reaction time (B: 1.0–5.0 h), catalyst concentration (C: 1–10%), and glycerol:acetic acid molar ratio (D: 1:3–1:7). The response variables for the optimization were glycerol conversion and selectivity for MAG, DAG, and TAG of the reaction products. Table 1 presents the independent variables, along with their codifications and values.

**Table 1:** Independent Variables, Coded Levels, and Evaluated Intervals in the Experimental Design

			Level of Factors		
Independent Variables	Unit	Coding	1	2	3
Reaction temperature	°C	A	60	90	120
Reaction time	h	B	1	3	5
Catalyst concentration	%	C	1	5.5	10
Molar ratio glycerol:acetic acid	mol/mol	D	1:3	1:5	1:7

To determine the number of experimental trials to be performed, eq 3 was employed.

3 N=(L−1)×P+1

where *N* represents the number of experimental trials, *L* represents the levels studied, and *P* represents the independent variables evaluated.

Thus, on the basis of this equation, nine experimental trials were defined, corresponding to the Taguchi L9 orthogonal design. Finally, it should be noted that all results obtained for glycerol conversion and MAG, DAG, and TAG selectivity of the reaction products after the nine experiments were statistically treated by using Minitab 18 software.

### 2.7 Signal-to-Noise Ratio (SNR) and Analysis of Variance (ANOVA)

In Taguchi orthogonal design, the signal-to-noise ratio (SNR) aims to ensure greater robustness and efficiency by reducing sensitivity to external factors, as it quantifies the relationship between the mean of the desired response (signal) and the undesirable variability (noise), expressing this relationship in a single value.^23^ Furthermore, depending on the intended objective for the response, the signal-to-noise ratio can be obtained through three different functions: larger-the-better (LTB), indicated for maximizing results; smaller-the-better (STB), ideal for minimization; and nominal-the-better (NTB), used when seeking an ideal value within a specific range.^33^ In the present study, the larger-the-better (LTB) function was adopted, since the objective is to maximize glycerol conversion and MAG, DAG, and TAG selectivity. The signal-to-noise ratios (SNR) for the study were determined from eq 4.

4 $$LTB=-\log\left(\sum_{j=1}^{n}\;\frac{1}{Y_{j}^{2}}\right)$$

where

5 $$y_{i}=\frac{1}{n}\left(\sum_{j=1}^{n}\;y_{\mathrm{ij}}\right)$$

6 $$S_{i}^{2}=\frac{1}{n-1}\left(\sum_{j=1}^{n}\;y_{\mathrm{ij}}-{\mathrm{y̅}}_{i}\right)$$

where

*y*_*i*_ is the measured value for the response (glycerol conversion, MAG, DAG, or TAG selectivity);

*S*_*i*_^2^ is the variance of the results;

*n* is the total number of experimental trials;

*i* is the experimental run index;

*j* is the number of repetitions of the experimental run.

Finally, it should be noted that the SNR is a tool for adjusting the results obtained for the response variables; however, it does not allow quantifying the exact effect and contribution of each variable to the acetylation reaction, making it necessary to perform analysis of variance (ANOVA) of the results in order to verify the contribution of each independent variable to the obtained responses. The contribution of each variable to the reaction was determined using eqs 7–9.

7 $$contribution\;factor\;\left(\%\right)=\frac{{SS}_{f}}{{SS}_{T}}$$

8 $${SS}_{f}=\sum_{j=1}^{n}\;n{\left[{\left({SNR}_{L}\right)}_{\mathrm{fj}}-{SNR}_{T}\right]}^{2}$$

9 $${SS}_{T}=\sum_{i=1}^{n}\;n{\left({SNR}_{i}-{SNR}_{T}\right)}^{2}$$

where

SS_f_ and SS_T_ are the sum of squares of factor *f* and the total sum of squares of all variables, respectively.

SNR_T_ represents the total mean SNR;

SNR_L_ expresses the SNR value for each level.

*r* indicates the number of repetitions (or runs) for factor *f*.

## 3 Results and Discussion

### 3.1 Characterization of the Materials

Surface acidity, an essential property for catalytic solids, quantifies the amount of acid sites present on the surface of these materials, which are responsible for mediating ion exchange between reactants and, consequently, promoting product formation.^33^ Thus, the surface acidity values obtained for CM750 biochar, sulfonated CMS750 biochar, NiFe_2_O_4_, and CMS750-NiFe_2_O_4_ catalyst are presented in Table 2.

**Table 2:** Surface Acidity of Synthesized Materials

Materials	Surface Acidity (mmol H^+^ g^–1^)
Biochar CM750	0.201 ± 0.002
NiFe_2_O_4_	0.152 ± 0.005
Sulfonated biochar CMS750	1.210 ± 0.022
Catalyst CMS750-NiFe_2_O_4_	1.368 ± 0.028

The obtained results show that CM750 biochar and NiFe_2_O_4_ exhibit a slightly acidic character, with values of 0.201 ± 0.002 and 0.152 ± 0.005, respectively. These values evidence the minimal presence of acid sites on the surface of CM750 and NiFe_2_O_4_, which is desirable given that these materials act as support and magnetic phase of the catalyst, i.e., they are expected to be chemically inert during the reaction, not contributing significantly to the catalytic activity of the CMS750-NiFe_2_O_4_ catalyst.^34,35^ On the other hand, the sulfonated CMS750 biochar presented significantly higher acidity, 1.210 ± 0.022, which can be related to the incorporation of sulfonic groups onto its surface due to the sulfonation process.^28,36^ Finally, the surface acidity value determined for the CMS750-NiFe_2_O_4_ catalyst was 1.368 ± 0.028, evidencing that the incorporation of sulfonic groups during sulfonation was efficient, since this value is approximately seven times higher than those obtained for CM750 biochar and NiFe_2_O_4_. It is also noteworthy that the incorporation of nickel ferrite did not promote a significant increase in the material’s acidity, indicating that the density of acid sites is directly associated with the sulfonic groups introduced into the carbonaceous matrix. Such an increase in acid site density is desirable, as they are fundamental for promoting higher glycerol conversions into acetals.^37,38^

Understanding the thermal behavior and stability of materials is fundamental to materials science, as these properties directly influence several synthesis processes. Thus, murici seed, CM750 biochar, uncalcined NiFe_2_O_4_, and CMS750-NiFe_2_O_4_ catalyst were analyzed by thermogravimetric analysis (TGA), and Figure 2 shows the TGA and DTG curves obtained for the materials.

**Figure 2:**
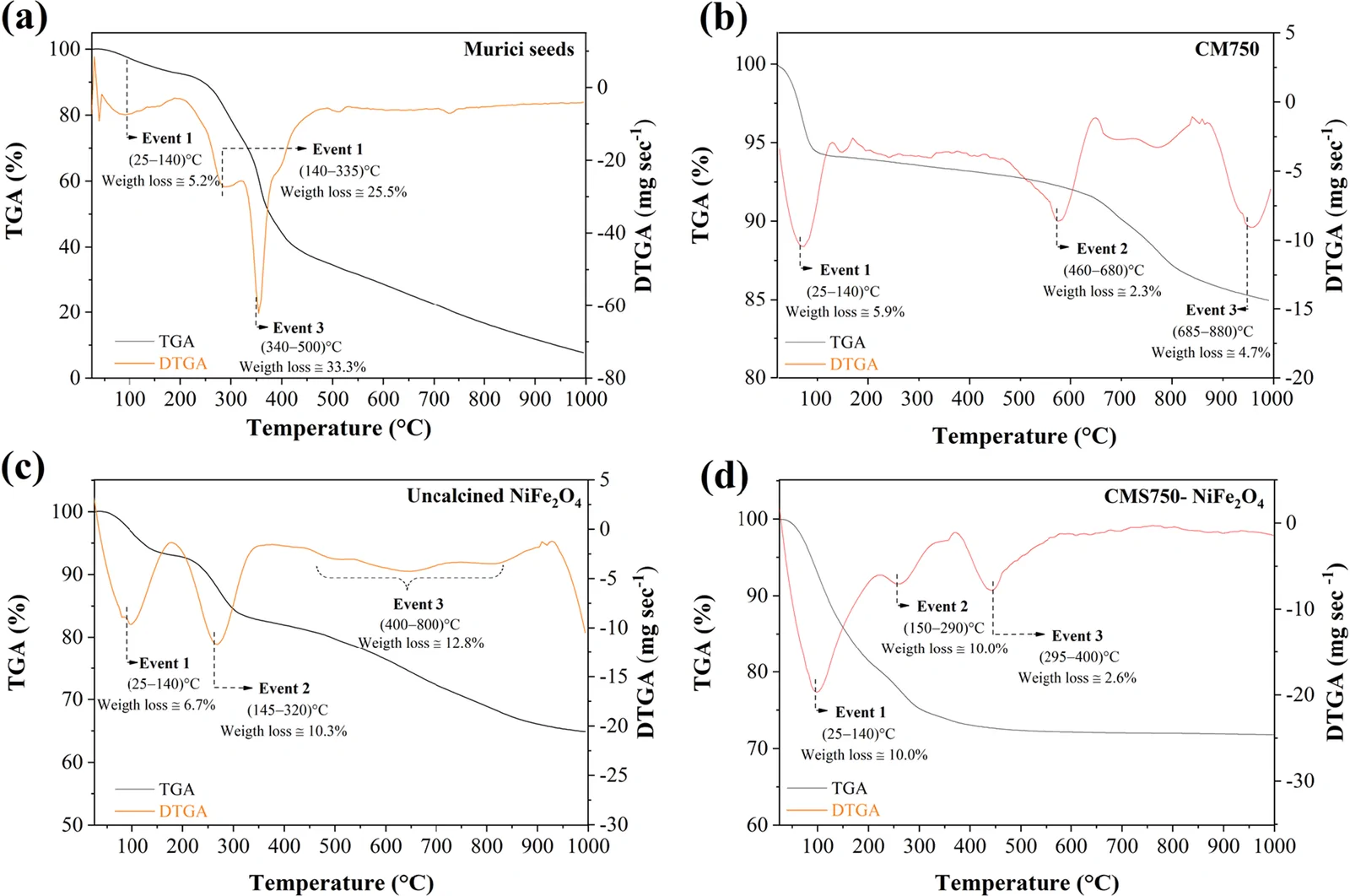
Thermogravimetric analysis of (a) muruci seed, (b) biochar CM750, (c) uncalcined NiFe_2_O_4_, and (d) catalyst CMS750-NiFe_2_O_4_.

Analysis of the results illustrated in Figure 2 shows that all materials, murici seed, CM750 biochar, NiFe_2_O_4_, and CMS750-NiFe_2_O_4_ catalyst, exhibit their first mass loss event within a common temperature range of 25–140 °C. This event may be related to the desorption of physically adsorbed water.^39,40^ Figure 2(a) presents the TGA and DTG curves obtained for murici seed, in which two mass loss events are observed after 140 °C: event 2 occurring in the temperature range of 140–335 °C and event 3 occurring in the range of 340–500 °C. These events can be related to the decomposition of lignocellulosic components with lower thermal stability (hemicellulose and cellulose) and to the decomposition of the lignocellulosic component with higher thermal stability (lignin), respectively.^41–43^

The TGA and DTG curves for CM750 biochar are shown in Figure 2(b). Event 2, which comprises the temperature range of 460–680 °C with a mass loss of 2.3%, can be associated with the pyrolysis of hemicellulose remaining from the carbonization process of murici seed.^16,44^ Event 3, with a mass loss of 4.7% occurring in the temperature range of 685–880 °C, can be attributed to the thermal decomposition of lignin structures that were not completely carbonized.^45^ Finally, it is worth noting that the CM750 biochar exhibits high thermal stability and that the carbonization temperature of 750 °C adopted in this study is suitable, since above 700 °C no significant mass loss events occur and the material retains approximately 85% of its initial mass.

Figure 2(c) presents the TGA and DTG curves for the NiFe_2_O_4_ precursor sample, prior to calcination at 800 °C for 2 h. Event 2, with a mass loss of 10.3% in the temperature range of 145–320 °C, is attributed to the decomposition of nitrate ions (NO_3_^–^) derived from the nickel nitrate hexahydrate precursor used as the nickel source in the ferrite synthesis (Vargas-Bustamante et al.;^46^ Idehara et al.^47^). Event 3, observed between 423 and 800 °C with a mass loss of 12.8%, is associated with the decomposition of residual species from the synthesis and with the temperature range in which the NiFe_2_O_4_ spinel phase is formed (Akhtar et al.;^48^ Hiremath et al.^49^). This behavior confirms that the calcination temperature employed is suitable for obtaining nickel ferrite.

Finally, Figure 2(d) presents the TGA and DTG curves obtained for the CMS750-NiFe_2_O_4_ catalyst. Event 2, which exhibits a mass loss of 10.0% and comprises the temperature range of 190–290 °C, can be attributed to the thermal degradation of sulfonic groups incorporated into the biochar structure during the sulfonation process.^50,51^ Event 3, observed in the temperature range of 295–400 °C with a mass loss of 2.6%, can be attributed to the decomposition of lignocellulosic compounds remaining from the carbonization process of murici seed.^52^ It is noteworthy that the thermal analysis results of the CMS750-NiFe_2_O_4_ catalyst demonstrate that the catalyst can be employed without the risk of thermal degradation in the glycerol acetylation reaction, since the temperature range adopted in this study is 60–120 °C.

In order to understand and evaluate the crystalline structure of the synthesized materials, X-ray diffraction analysis was performed. The diffractograms obtained for CM750 biochar, NiFe_2_O_4_, and the CMS750-NiFe_2_O_4_ catalyst are illustrated in Figure 3.

**Figure 3:**
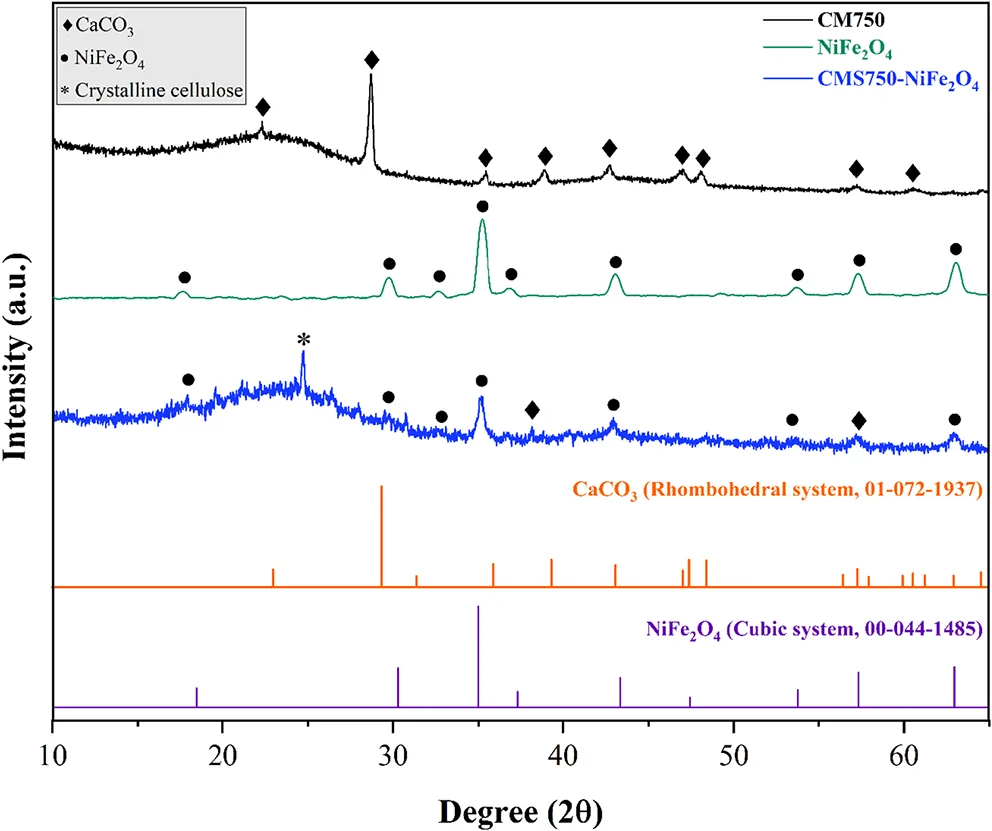
X-ray diffractograms for biochar CM750, NiFe_2_O_4_, and catalyst CMS750-NiFe_2_O_4_.

In the diffractogram of CM750 biochar (black line), two characteristic halos of carbon-rich materials are observed, located in the 2θ regions of 15–30° and 40–50°, which can be attributed to the (002) and (101) crystallographic planes, typical of amorphous carbon structures composed of aromatic carbon sheets.^39,51^ Finally, the diffractogram of CM750 biochar shows the presence of an inorganic calcium carbonate (CaCO_3_) phase in the biochar structure, with peaks located at 2θ = 28.6°, 35.1°, 38.5°, 42.4°, 47.6°, 57.2°, and 60.5°, associated with the rhombohedral polymorphic structure of CaCO_3_, according to JCPDS card No. 01-072-1937. The diffractogram corresponding to NiFe_2_O_4_ (green line) exhibits characteristic peaks of the NiFe_2_O_4_ spinel phase at 18.5° (111), 30.3° (220), 35.7° (311), 37.3° (222), 43.4° (400), 53.8° (422), 57.4° (511), and 63.1° (440), in accordance with standard JCPDS card No. 00-044-1485, indicating the successful synthesis of nickel ferrite by coprecipitation.

Finally, in the diffractogram corresponding to the CMS750-NiFe_2_O_4_ catalyst (blue line), a well-defined crystallographic peak is observed at 2θ = 22.5°, attributed to the presence of crystalline cellulose structures in the material.^43^ It is also possible to verify a reduction in the intensity of the carbon halos located in the 2θ regions of 15–30° and 40–50° when compared to CM750 biochar.^22^ Furthermore, the inorganic CaCO_3_ phase exhibited a decrease in the magnitude of the peaks located at 2θ = 42.4° and 57.2°, attributed to the rhombohedral polymorphic structure of CaCO_3_, according to JCPDS card No. 01-072-1937. These structural changes in the material can be linked to the sulfonation process of CM750 biochar with concentrated sulfuric acid, which promotes the breaking of bonds between elements present in the material, causing weakening of the carbon sheets, increased disorder of the carbonaceous structures, as well as modification and reduction of the crystalline structure of CaCO_3_ in the material.^37,50^ Finally, the diffractogram displays characteristic diffraction peaks of NiFe_2_O_4_ located at 18.5° (111), 30.3° (220), 35.7° (311), 37.3° (222), 43.4° (400), 53.8° (422), 57.4° (511), and 63.1° (440), according to standard JCPDS card No. 00-044-1485, indicating the successful impregnation of the magnetic phase into the material.

Figure 4 presents the FT-IR spectra for CM750 biochar, NiFe_2_O_4_, and the CMS750-NiFe_2_O_4_ catalyst. In the spectrum corresponding to CM750 biochar (black line), characteristic bands of carbonized lignocellulosic matrices are observed, such as at 2335 cm^–1^, related to the normal vibrational mode (NVM) of the C=C bond stretching, characteristic of aromatic rings, and at 2190 cm^–1^, attributed to the NVM of the C≡C bond stretching of alkyl groups.^7,21^ The presence of vibrational bands at 1704 and 1593 cm^–1^ is also noted, related to the NVM of C–O and C=C bond stretching, attributed to the presence of carboxylic groups and aromatic rings, respectively.^10,50^ Furthermore, the spectrum evidences a vibrational band at 874 cm^–1^, referring to the out-of-plane NVM of the C=O bond, which is attributed to the presence of the carbonate group (CO_3_^2–^), reinforcing the presence of CaCO_3_ structures in CM750 biochar, as indicated by XRD analysis.^50,53^ Finally, the spectrum also presents a vibrational band at 741 cm^–1^, associated with the angular deformation of the =C–H bond, characteristic of aromatic structures formed during the biomass carbonization process.^28^

**Figure 4:**
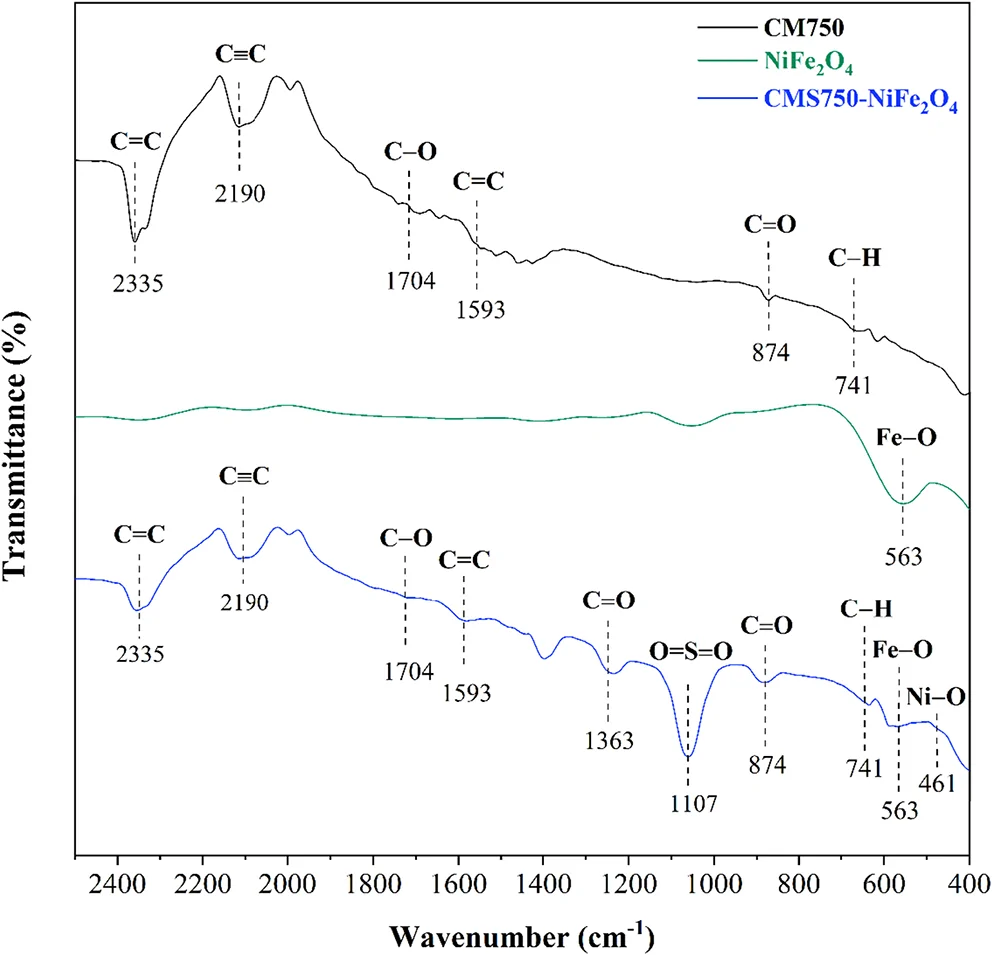
FT-IR spectra for CM750 biochar, NiFe_2_O_4_, and catalyst CMS750-NiFe_2_O_4_.

In the spectrum corresponding to NiFe_2_O_4_ (green line), a characteristic vibrational band of inverse spinel-type ferrites is observed at 563 cm^–1^, which can be related to the normal vibrational mode (NVM) of the metal (Ni and Fe) oxygen bonds present in the octahedral and tetrahedral sites, respectively. Finally, the spectrum of the CMS750-NiFe_2_O_4_ catalyst (blue line) exhibits NVM bands typical of materials rich in carbonized lignocellulosic compounds.^54,55^ Furthermore, the presence of the out-of-plane NVM of the C=O bond at 874 cm^–1^ is noted, which is attributed to the carbonate group (CO_3_^2–^).^56^ Additionally, it is also possible to observe the appearance of a band located in the region of 1107 cm^–1^, attributed to the symmetric stretching NVM of the O=S=O bond, characteristic of the presence of sulfonic groups in the material.^3,54^ Two bands are also observed at 563 and 461 cm^–1^, characteristic of the Fe–O and Ni–O bonds located in the octahedral and tetrahedral sites, typical of the presence of the inverse spinel phase of nickel ferrite.^2,57^ It is noteworthy that the presence of these three vibrational bands indicates the success of the sulfonation and magnetic phase impregnation processes employed in the synthesis of the CMS750-NiFe_2_O_4_ catalyst.

Figure 5 presents the N_2_ adsorption–desorption isotherms obtained by the Brunauer–Emmett–Teller (BET) method for CM750 biochar and the CMS750-NiFe_2_O_4_ catalyst. Figure 5(a) and 5(b) shows that both materials exhibit type IV isotherms, characteristic of mesoporous solids. A hysteresis loop is also observed in the relative pressure range (*P*/*P*_0_) between approximately 0.4 and 1.0, associated with capillary condensation in the mesopores, further confirming the mesoporous nature of the materials.^50,52^ Figure 5(c) and 5(d) displays the pore size distributions obtained by the BJH method. The results indicate that both CM750 biochar and the CMS750-NiFe_2_O_4_ catalyst exhibit a predominant pore distribution in the mesoporous range (2–50 nm).^36,39^ After the sulfonation and impregnation with NiFe_2_O_4_, a reduction in pore volume is observed, suggesting that part of the mesopores was partially occupied by sulfonic groups and the magnetic phase during catalyst synthesis, without, however, promoting significant changes in the average pore diameter.

**Figure 5:**
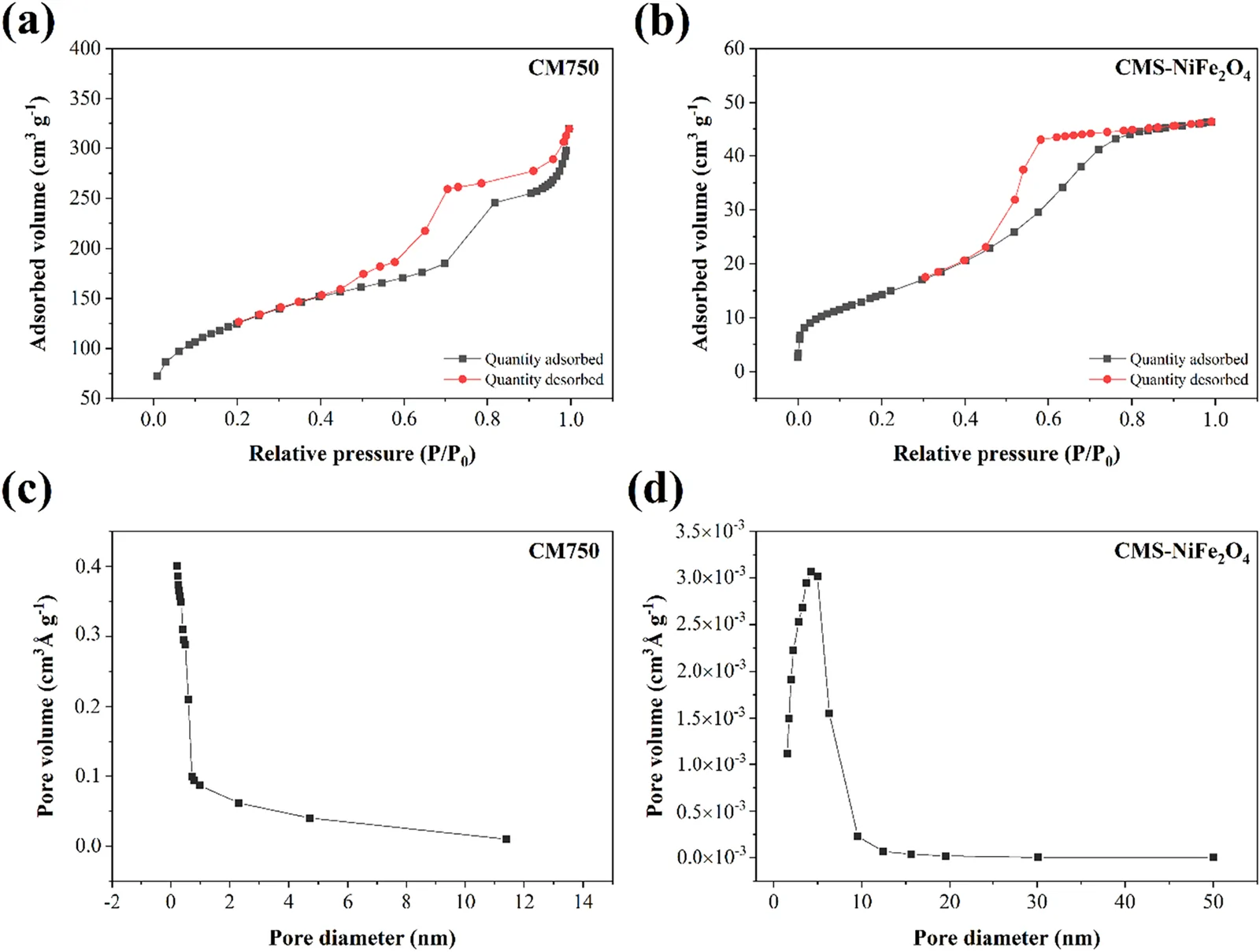
N_2_ adsorption and desorption isotherms for (a) CM750 biochar and (b) CMS750-NiFe_2_O_4_ catalyst. Average pore size distribution for (c) CM750 biochar and (d) CMS750- NiFe_2_O_4_ catalyst.

The textural parameters obtained from the BET analyses are presented in Table 3. CM750 biochar exhibited a high specific surface area (451.16 m^2^ g^–1^) and total pore volume (0.400 cm^3^ g^–1^), evidencing a well-developed mesoporous structure. After the sulfonation and impregnation with NiFe_2_O_4_, the specific surface area decreased to 111.55 m^2^ g^–1^, while the total pore volume was reduced to 0.160 cm^3^ g^–1^. This reduction can be attributed to the incorporation of sulfonic groups and the deposition of the magnetic phase (NiFe_2_O_4_) onto the biochar surface, as well as to the partial blockage of porous channels, which reduced the area accessible to N_2_ adsorption. In contrast, the average pore diameter underwent only a slight change, from 55.69 to 52.98 Å (5.57 and 5.30 nm, respectively), indicating that impregnation preserved the mesoporous structure of the biochar, mainly promoting partial pore occupation without significantly altering its architecture.

**Table 3:** Textural Properties of CM750 Biochar and Catalyst CMS750-NiFe_2_O_4_

Sample	Surface Area (m^2^ g^–1^)	Pore Size (cm^3^ g^–1^)	Average Pore Size (Å)
CM750 biochar	451.157	0.400	55.693
catalyst CMS750-NiFe_2_O_4_	111.547	0.160	52.975

The surface micrographs obtained for CMS750 biochar, NiFe_2_O_4_, and CMS750-NiFe_2_O_4_ catalyst are presented in Figure 6, revealing the morphological characteristics of each sample. The micrographs corresponding to CM750 biochar (Figure 6(a), 6(b), and 6(c)) reveal an irregular surface morphology, with a broad and highly dispersed porous structure throughout the material, formed through the release of gases during the carbonization process of murici biomass.^15,42^ Figure 6(d), 6(e), and 6(f), referring to NiFe_2_O_4_, shows an irregular surface with agglomerates of different sizes and absence of defined morphology.^24,57^ In turn, the micrographs of the CMS750-NiFe_2_O_4_ catalyst (Figure 6(g), 6(h), and 6(i)) show significant morphological changes compared to those observed for CM750 biochar. A surface with a brittle appearance is observed, which can be related to the sulfonation process since concentrated sulfuric acid acts as an oxidizing agent.^58^ Furthermore, it is also possible to note a partial coating of the surface, which may be related to the deposition of the NiFe_2_O_4_ magnetic phase onto the CM750 biochar surface.

**Figure 6:**
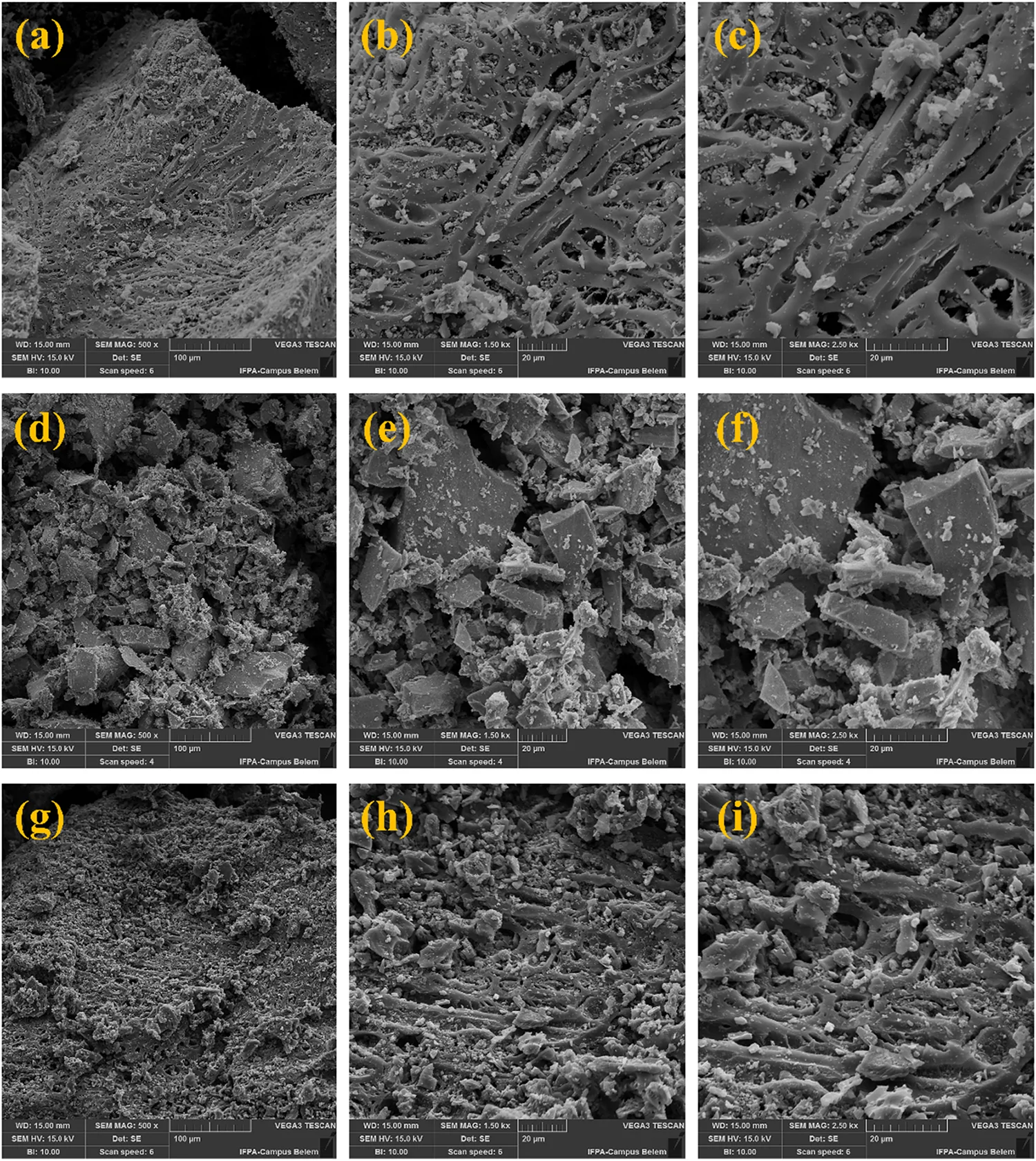
Micrographs for (a) CM750 biochar 500× magnification, (b) CM750 biochar 1500× magnification, (c) CM750 biochar 2500× magnification, (d) NiFe_2_O_4_ 500× magnification, (e) NiFe_2_O_4_ 1500× magnification, (f) NiFe_2_O_4_ 2500× magnification, (g) catalyst CMS750-NiFe_2_O_4_ 500× magnification, (h) catalyst CMS750-NiFe_2_O_4_ 1500× magnification, and (i) catalyst CMS750-NiFe_2_O_4_ 2500× magnification.

Figure 7 presents the elemental composition and EDS mapping of the surface of CM750 biochar and CMS750-NiFe_2_O_4_ catalyst. For CM750 biochar (Figure 7(a) and 7(b)), the analysis reveals a high carbon (C) content (92.12% ± 0.155), with uniform distribution, and a low oxygen (O) content (6.97% ± 0.176), the latter exhibiting a predominantly homogeneous distribution with small agglomerates. These carbon (C) and oxygen (O) concentrations are characteristic of the synthesis process, via carbonization of murici seed under an inert N_2_ atmosphere. The analysis also detects the presence of calcium (Ca) (0.87% ± 0.105), whose spatial distribution is similar to that of oxygen. This result corroborates the XRD and FT-IR data, which indicate the presence of CaCO_3_ in the material structure.

**Figure 7:**
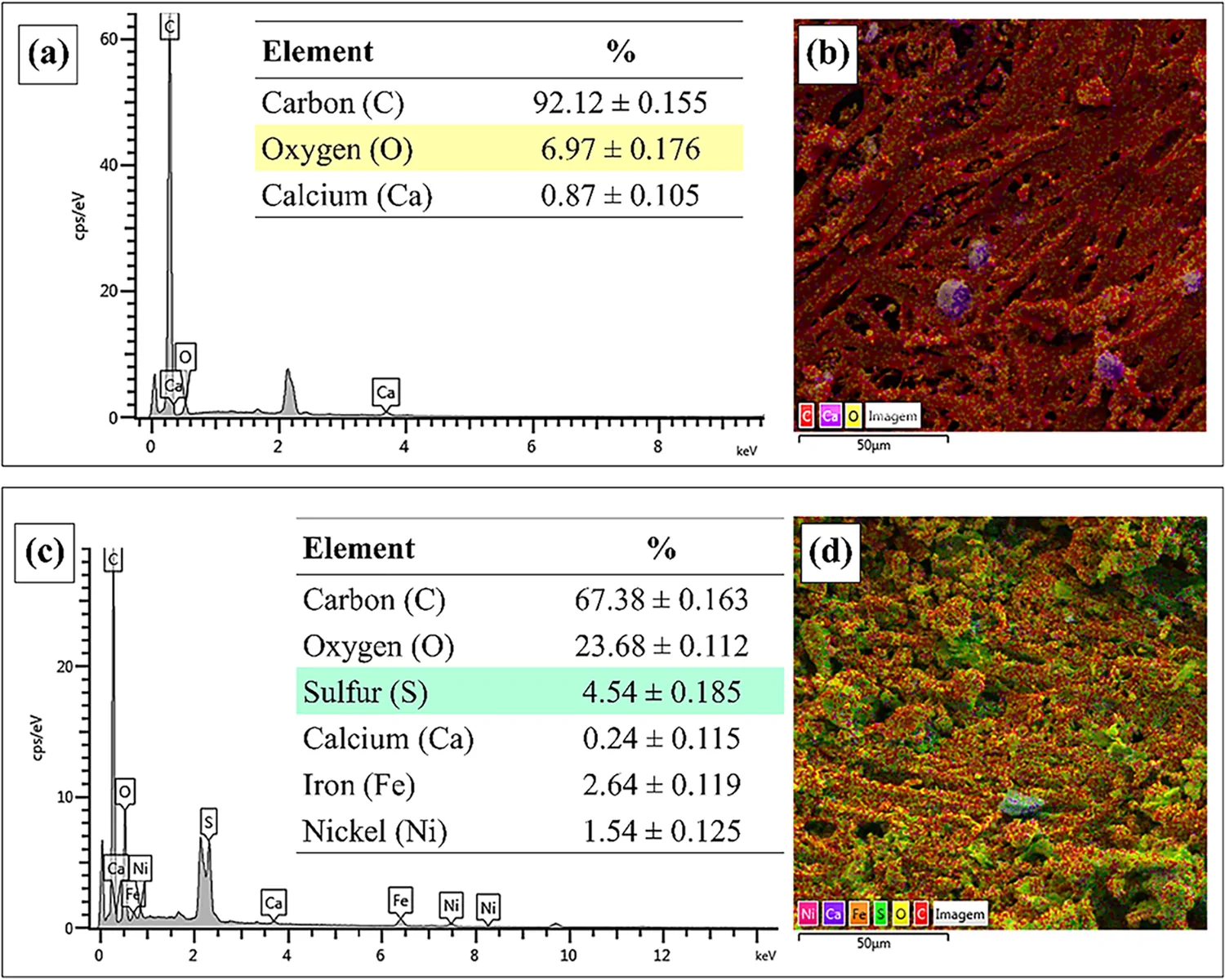
(a) EDS spectrum of CM750 biochar, (b) surface elemental mapping of CM750 biochar, (c) EDS spectrum of catalyst CMS750-NiFe_2_O_4_, and (d) surface elemental mapping of catalyst CMS750-NiFe_2_O_4_.

Figure S1 presents the EDS analysis and elemental mapping of the synthesized NiFe_2_O_4_. The elemental composition (Figure S1­(a)) reveals contents of 55.22% ± 0.175 nickel (Ni) and 26.1% ± 0.135 iron (Fe) on the material surface. These values are close to the stoichiometric ratio of 2:1 between Fe^3+^ and Ni^2+^ used in the ferrite synthesis. The elemental mapping (Figure S1­(b)) further evidences the homogeneous distribution of Ni, Fe, and oxygen (O) on the surface, confirming the effectiveness of the coprecipitation method employed.

EDS analysis and elemental mapping of the CMS750-NiFe_2_O_4_ catalyst (Figure 7(c) and 7(d)) reveal a sulfur (S) content of 4.54% ± 0.185, homogeneously distributed on its surface, confirming the effectiveness of sulfonation in incorporating sulfonic groups into the biochar structure. Compared to CM750, a reduction of ∼24.75% in carbon (C) content and an increase of ∼16.71% in oxygen (O) content are observed. This change is a direct consequence of the sulfonation process, which promotes the formation of oxygenated groups in the material structure.^37^ The contents of Ni (1.54% ± 0.125) and Fe (2.64% ± 0.185) are also detected in a proportion close to the stoichiometric ratio (2:1), attesting to the successful incorporation of the NiFe_2_O_4_ magnetic phase. Finally, the presence of calcium (0.25% ± 0.115) is in agreement with the XRD and FT-IR analyses, which indicated the presence of CaCO_3_ in the compositions of CM750 biochar and CMS750-NiFe_2_O_4_ catalyst.

Figure 8 presents the hysteresis curves obtained for NiFe_2_O_4_ and the CMS750-NiFe_2_O_4_ catalysts, as well as the separation process of the catalyst from the reaction medium by applying a magnetic field.

**Figure 8:**
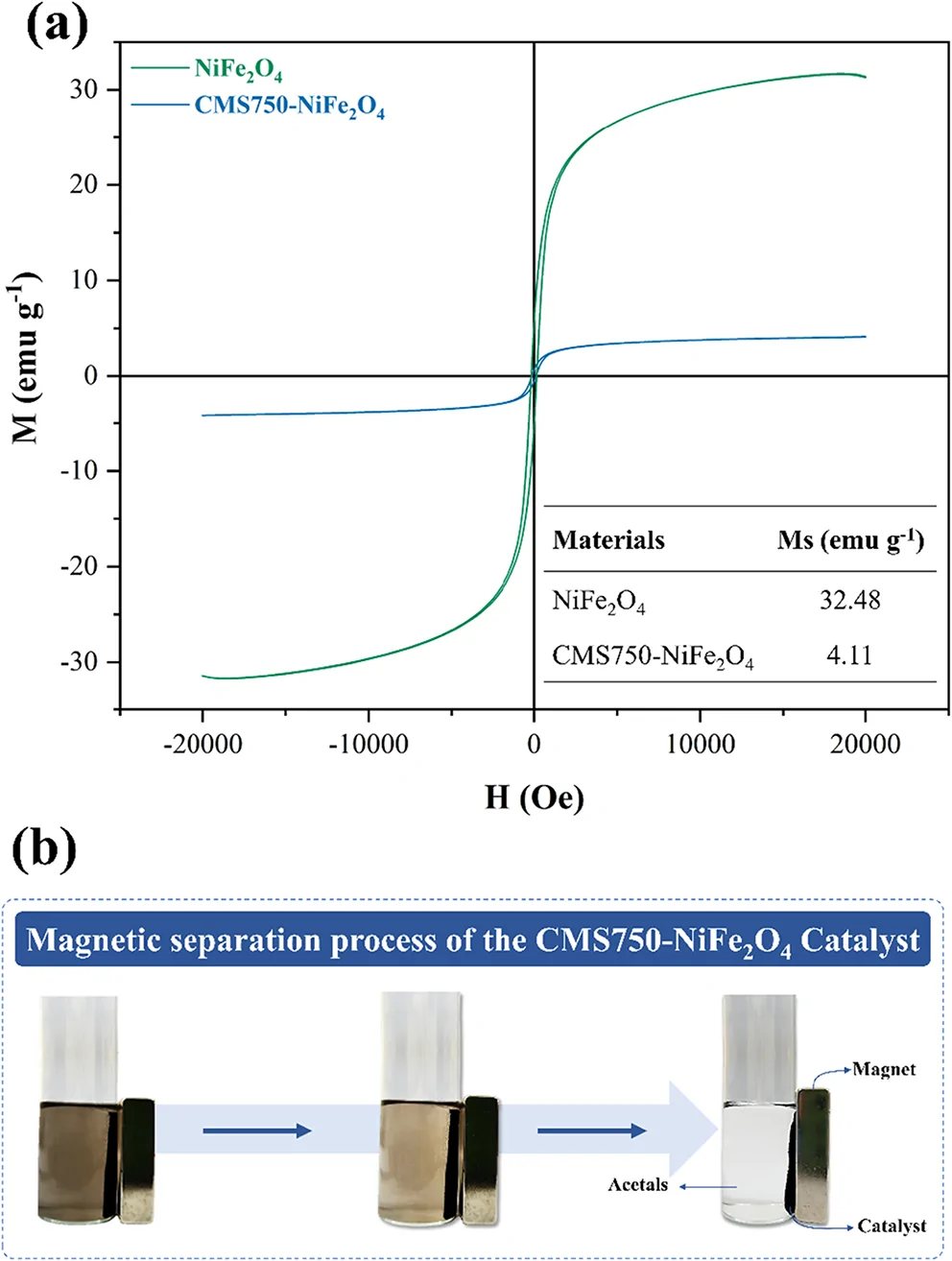
(a) Hysteresis curves for NiFe_2_O_4_ and CMS750-NiFe_2_O_4_ catalyst and (b) magnetic separation process.

The results presented in Figure 8 show that NiFe_2_O_4_ exhibits a saturation magnetization (Ms) of 32.48 emu g^–1^, a value similar to that reported by Talebi^59^ (Ms of 34.0 emu g^–1^) when investigating the photocatalytic properties of NiFe_2_O_4_ synthesized via the sol-gel method. Xu et al.^60^ studied the synthesis of magnetic composites applied to herbicide removal from water and observed an Ms value of 32.1 emu g^–1^ for NiFe_2_O_4_ synthesized via the hydrothermal method. Similarly, Gonçalves et al.,^24^ employing the coprecipitation method in the production of acid magnetic catalysts based on NiFe_2_O_4_ for biodiesel synthesis, reported a value of 36.3 emu g^–1^. The value recorded in this study, together with the other characterizations performed (SA, TGA, XRD, FT-IR, and EDS), attests to the successful obtention of nickel ferrite through the coprecipitation method.

In turn, the CMS750-NiFe_2_O_4_ catalyst exhibited an Ms of 4.11 emu g^–1^, representing a reduction of 87.34% compared to the pure magnetic phase (NiFe_2_O_4_). This drastic decrease is expected and attributed to the final composition of the catalyst, consisting mainly (∼90 wt %) of nonmagnetic species (sulfonated biochar). Analogous behaviors have been reported in the literature by Chellappan et al.,^61^ who observed Ms of 29.25 emu g^–1^ for pure Fe_3_O_4_ and 11.16 emu g^–1^ after its impregnation onto a sulfonated biochar, and by Chutia et al.,^62^ who reported values of 13.8 and 11.32 emu g^–1^ for similar situations. In both studies, the authors validated the applicability of the magnetic catalysts in transesterification reactions. It is important to highlight that, even with the observed reduction, the Ms of the CMS750-NiFe_2_O_4_ catalyst is sufficiently high to allow its rapid recovery by applying an external magnetic field, as illustrated in Figure 8(b).

### 3.2 Statistical Model Analysis and Study of the Influence of Reaction Variables on Glycerol Conversion and MAG, DAG, and TAG Selectivity

The Taguchi orthogonal design (L9), with nine experimental trials, was adopted to optimize the acetylation reaction of glycerol with acetic acid. In order to maximize glycerol conversion and MAG, DAG, and TAG selectivity, the larger-the-better (LTB) function was adopted for the study. The percentage values of glycerol conversion and MAG, DAG, and TAG selectivity obtained in the nine experimental trials, together with the respective signal-to-noise ratio (SNR) values and the overall mean (SNR_T_), are presented in Table 4.

**Table 4:** Glycerol Conversion, Selectivity to MAG, DAG, and TAG, and Corresponding Signal-to-Noise Ratio (SNR, dB) and SNR_T_ Values for the L9 Taguchi Design^a^

						Conversion (%)		Selectivity (%)					
Experiment	Run Order	*A*	*B*	*C*	*D*	Glycerol	SNR (dB)	MAG	SNR (dB)	DAG	SNR (dB)	TAG	SNR (dB)
**1**	3	1	1	1	1	36.98	31.35	85.82	38.67	9.08	19.16	5.10	14.15
**2**	5	1	2	2	2	75.08	37.51	66.37	36.43	25.56	28.15	8.07	18.13
**3**	2	1	3	3	3	96.97	39.73	24.39	27.74	58.69	35.37	16.92	24.56
**4**	6	2	1	2	3	90.75	39.15	32.10	30.13	48.42	33.70	19.48	25.79
**5**	1	2	2	3	1	88.01	38.89	49.36	33.86	35.11	30.90	15.53	23.82
**6**	4	2	3	1	2	90.90	39.17	34.44	30.74	49.13	33.82	16.43	24.31
**7**	8	3	1	3	2	99.33	39.94	15.58	23.85	53.50	34.56	30.92	29.80
**8**	9	3	2	1	3	95.46	39.59	18.26	25.23	58.26	35.30	25.08	27.98
**9**	7	3	3	2	1	98.94	39.90	18.93	25.54	55.82	34.93	25.25	28.04
**SNR**_**T**_ **– Overall mean**						–	38.96	–	38.67	–	28.15	–	24.06

a SNR: signal-to-noise ratio expressed in decibels (dB), SNR_T_: overall mean signal-to-noise ratio; and *A*, *B*, *C*, and *D* denote reaction temperature, reaction time, catalyst loading, and glycerol:acetic acid molar ratio, respectively.

Analyzing the results presented in Table 4, it is observed that Experiment 1 exhibits relatively low SNR values for glycerol conversion (31.35 dB), DAG selectivity (19.16 dB), and TAG selectivity (14.15 dB), associated with a glycerol conversion of only 36.98%, and selectivities of 9.08% for DAG and 5.10% for TAG. These results demonstrate that Experiment 1 shows high sensitivity to variations and, therefore, unfavorable reaction conditions (60 °C, 1 h, 1:3, and 1%) for the acetylation reaction, since the reaction is endothermic and requires higher temperatures,^12,30^ longer reaction times to favor interaction between reactants and the catalyst active sites,^4,19^ an excess of acetic acid to shift the equilibrium, and sufficient acid sites to promote higher conversion values and selectivity for the more substituted products.^6,15^ It is also noteworthy that the experimental conditions favor only MAG selectivity (85.82%), since the obtained SNR was 38.67 dB.

On the other hand, experiments 3, 7, 8, and 9 demonstrated SNR values above 39 dB, indicating greater stability and robustness for the acetylation reaction. In these experiments, high glycerol conversions were observed with values of 96.97%, 99.33%, 95.46%, and 98.94%, respectively. An increase in SNR values for the selectivity of the more substituted reaction products was also verified: for DAG, the SNR ratio was above 34 dB, and for TAG, above 24.5 dB, with a reduction in SNR for MAG below 25 dB. These results reinforce that increasing the reaction parameters employed significantly favors catalytic performance, since higher SNR values indicate lower experimental variability and, consequently, result in a more efficient process.^64^

Furthermore, it is noteworthy that the experimental trials with higher SNR suggest that the variables of reaction temperature (factor A) and glycerol:acetic acid molar ratio (factor *D*) exert greater influence on glycerol conversion and on MAG, DAG, and TAG selectivity. Finally, the overall mean signal-to-noise ratio (SNR_T_) values of 38.96 dB for glycerol conversion, 38.67 dB for MAG, 28.15 dB for DAG, and 24.06 dB for TAG indicate a high overall quality of the glycerol acetylation reaction with low experimental variability among the results.^63^

In order to determine the order of influence of the variables reaction temperature (A), reaction time (B), catalyst concentration (C), and glycerol:acetic acid molar ratio (D) on glycerol conversion and MAG, DAG, and TAG selectivity, the DSNR_L_ parameter was used. DSNR_L_ is obtained by the difference between the maximum and minimum SNR_L_ values for each evaluated variable. Tables 5, 6, 7, and 8 present the SNR_L_ values determined for glycerol conversion and for MAG, DAG, and TAG selectivity, respectively.

**Table 5:** Signal-to-Noise Ratio (SNR_L_, dB) Values at Each Factor Level for Glycerol Conversion and Corresponding Factor Ranking^a^

	SNR Corresponding to the Biodiesel Ester Content			
Level	*A*	*B*	*C*	*D*
1	69.68	75.69	75.45	74.64
2	89.89	86.18	88.26	88.44
3	97.91	95.60	94.77	94.39
DSNR_L_	28.23	19.92	20.30	19.75
Rank	1	3	2	4

a SNR_L_, signal-to-noise ratio at each factor level (dB); DSNR_L_, difference between the maximum and minimum SNR_L_ values for each factor; *A*, *B*, *C*, and *D* correspond to reaction temperature, reaction time, catalyst loading, and glycerol:acetic acid molar ratio, respectively.

**Table 6:** Signal-to-Noise Ratio (SNR_L_, dB) Values at Each Factor Level for MAG Selectivity and Corresponding Factor Ranking^a^

	SNR Corresponding to the Biodiesel Ester Content			
Level	*A*	*B*	*C*	*D*
1	56.86	44.50	46.17	51.17
2	38.63	44.66	39.13	38.80
3	17.59	25.92	29.78	24.92
DSNR_L_	41.27	18.74	16.40	26.45
Rank	1	3	4	2

a SNR_L_: signal-to-noise ratio at each factor level (dB), DSNR_L_: difference between the maximum and minimum SNR_L_ values for each factor, and *A*, *B*, *C*, and *D* correspond to the reaction temperature, reaction time, catalyst loading, and glycerol:acetic acid molar ratio, respectively.

**Table 7:** Signal-to-Noise Ratio (SNR_L_, dB) Values at Each Factor Level for DAG Selectivity and Corresponding Factor Ranking^a^

	SNR Corresponding to the Biodiesel Ester Content			
Level	*A*	*B*	*C*	*D*
1	31.11	37.00	38.82	33.34
2	44.22	39.64	43.27	42.73
3	55.86	54.55	49.10	55.12
DSNR_L_	24.75	17.55	10.28	21.79
Rank	1	3	4	2

a SNR_L_: signal-to-noise ratio at each factor level (dB), DSNR_L_: difference between the maximum and minimum SNR_L_ values for each factor, and *A*, *B*, *C*, and *D* correspond to reaction temperature, reaction time, catalyst loading, and glycerol:acetic acid molar ratio, respectively.

**Table 8:** Signal-to-Noise Ratio (SNR_L_, dB) Values at Each Factor Level for TAG Selectivity and Corresponding Factor Ranking^a^

	SNR Corresponding to the Biodiesel Ester Content			
Level	*A*	*B*	*C*	*D*
1	18.95	23.25	22.15	22.01
2	24.64	23.32	23.99	24.09
3	28.61	25.64	26.07	26.12
DSNR_L_	9.66	2.39	3.92	4.11
Rank	1	4	3	2

a SNR_L_, signal-to-noise ratio at each factor level (dB); DSNR_L_, difference between the maximum and minimum SNR_L_ values for each factor; *A*, *B*, *C*, and *D* correspond to reaction temperature, reaction time, catalyst loading, and glycerol:acetic acid molar ratio, respectively.

Analysis of Table 5 demonstrates that reaction temperature (A) is the factor with the greatest influence on glycerol conversion, presenting a DSNR_L_ value of 28.23 dB, which reinforces that temperatures above 90 °C favor increased glycerol conversion into acetals. The second most influential variable on glycerol conversion was catalyst concentration (C), with a DSNR_L_ value of 20.30 dB, indicating that catalyst concentrations above 5.5% lead to higher conversion values. In contrast, reaction time (B) and glycerol:acetic acid molar ratio (D) showed lower influence on conversion, with DSNR_L_ values of 19.92 and 19.75 dB, respectively, occupying the third and fourth positions in the order of influence, evidencing that their variations exert a less significant effect on glycerol conversion into acetals.

On the other hand, Tables 6 and 7, referring to MAG and DAG selectivity, respectively, also demonstrate that reaction temperature (A) is the most influential factor, with DSNR_L_ values of 41.27 and 24.75 dB, respectively. These results indicate that MAG selectivity is favored by temperatures around 60 °C, while DAG selectivity is favored by temperatures above 90 °C. The second most influential variable on MAG and DAG selectivity is the glycerol:acetic acid molar ratio (D), with DSNR_L_ values of 26.45 and 21.79 dB, indicating that glycerol:acetic acid molar ratios up to 1:4 favor MAG formation, whereas DAG formation is favored only by molar ratios above this value. Reaction time (B) and catalyst concentration (C) exhibit less significant influence on MAG and DAG selectivity, evidencing that their variations exert a less pronounced effect on the selectivity for these acetals.

Finally, analysis of Table 8, referring to TAG selectivity, demonstrates that reaction temperature (A) is the most influential factor, with a DSNR_L_ value of 24.75 dB, reinforcing that temperatures above 90 °C favor the formation of the most substituted product (TAG). The second most influential variable is the glycerol:acetic acid molar ratio (D), with a DSNR_L_ value of 21.79 dB, indicating that molar ratios above 1:4 promote increased selectivity for TAG. In contrast, reaction time (B) and catalyst concentration (C) showed lower influence, with DSNR_L_ values of 17.55 and 10.28 dB, respectively, occupying the third and fourth positions in the order of influence on TAG selectivity. These results evidence that variations in these two variables exert a less significant effect on TAG formation when compared to the other variables.

Furthermore, to quantify the extent of the influence of each variable and determine the percentage contribution of the independent variables to the response variables (glycerol conversion, MAG, DAG, and TAG selectivity), an analysis of variance (ANOVA) was performed on the obtained experimental results. Tables 8 and 9 present the ANOVAs for glycerol conversion and MAG, DAG, and TAG selectivity, respectively.

**Table 9:** ANOVA Results for Signal-to-Noise Ratios (SNR) Relating to Glycerol Conversion and Selectivity for MAG

	Factors	Sum of Squares	Degree of Freedom	Mean Square	Percentage Contribution (%)
**Glycerol**	A	21.86	2	10.93	36.68
	B	12.04	2	6.02	20.20
	C	12.97	2	6.48	21.77
	D	12.73	2	6.36	21.35
	Error	–	0	–	–
	Total	59.61	8	7.45	100.0

	Factors	Sum of Squares	Degree of Freedom	Mean Square	Percentage Contribution (%)
**MAG**	A	140.82	2	70.41	64.86
	B	23.90	2	11.95	11.01
	C	14.98	2	7.49	6.90
	D	37.43	2	18.71	17.23
	Error	–	0	–	–
	Total	217.15	8	27.60	100.0

Analysis of Table 9 reveals the percentage contribution of each variable in the experimental design. For glycerol conversion, the reaction temperature (A) was the most influential factor (36.68%), followed by catalyst concentration (C) with 21.77%. The glycerol:acetic acid molar ratio (D) and reaction time (B) showed similar contributions of 21.35% and 20.20%, respectively. Regarding MAG selectivity, the reaction temperature (A) also demonstrated dominance, with a contribution of 64.86%. The remaining variables had a lower influence on selectivity:molar ratio (D) with 17.23%, reaction time (B) with 11.01%, and catalyst concentration (C) with 6.90%.

Analysis of Table 10 allows identifying the relative contribution of each factor to product formation. For DAG formation, the reaction temperature (A) proved to be the most influential variable, with a percentage contribution of 39.42%, followed by the glycerol:acetic acid molar ratio (D) (30.73%), reaction time (B) (23.01%), and finally catalyst concentration (C) (6.83%). Concerning TAG formation, the reaction temperature (A) exhibited an even more predominant influence, accounting for 70.40% of the total contribution. The remaining variables had a significantly lower influence: molar ratio (D) (12.60%), catalyst concentration (C) (5.54%), and reaction time (B) (5.54%).

**Table 10:** ANOVA Results for Signal-to-Noise Ratios (SNR) Relating to Selectivity for DAG and TAG

	Factors	Sum of Squares	Degree of Freedom	Mean Square	Percentage Contribution (%)
**DAG**	A	919.92	2	459.96	39.42
	B	536.98	2	268.49	23.01
	C	159.38	2	79.69	6.83
	D	716.49	2	358.24	30.73
	Error	–	0	–	–
	Total	2332.77	8	291.60	100.0

	Factors	Sum of Squares	Degree of Freedom	Mean Square	Percentage Contribution (%)
**TAG**	A	141.45	2	70.72	70.40
	B	11.14	2	5.57	5.54
	C	23.02	2	11.51	11.45
	D	25.32	2	12.66	12.60
	Error	–	0	–	–
	Total	200.93	8	25.12	100.0

Furthermore, based on the ANOVAs, four regression models (eqs 10–11) were developed with a 95% confidence level to describe the behavior of the response variables, glycerol conversion and MAG, DAG, and TAG selectivity, in relation to the independent variables.

10 glycerolconversion(%)=−8.6+0.471A+4.98B+2.258C+4.94D

11 MAGselectivity(%)=157.3−0.688A−4.64B−1.822C−6.61D

12 DAGselectivity(%)=−40.07+0.412A+4.287B+1.142C+5.447D

13 TAGselectivity(%)=−18.18+0.284A+0.258B+0.621C+1.300D

Figure 9 displays the predicted versus observed value plots and the respective residual analyses for the statistical models of glycerol conversion and MAG selectivity. It is observed in Figure 9(a) and 9(c) that the experimental data for both responses are distributed close to the linear regression line, indicating a good predictive capability of the models within the 95% confidence interval. The coefficients of determination (*R* ^2^ and adjusted *R* ^2^) for the glycerol conversion and MAG selectivity models are 0.9577, 0.9517, 0.9650, and 0.9600, respectively. These high values indicate a strong correlation between experimental data and predictions, indicating that the proposed mathematical models are capable of explaining 95.17% and 96.00% of the result variability for conversion and MAG selectivity, respectively. Additionally, the residual analyses (Figure 9b and 9d) show that the errors are randomly distributed around the reference line (zero residual), validating the assumption of residual normality and the adequacy of the models.

**Figure 9:**
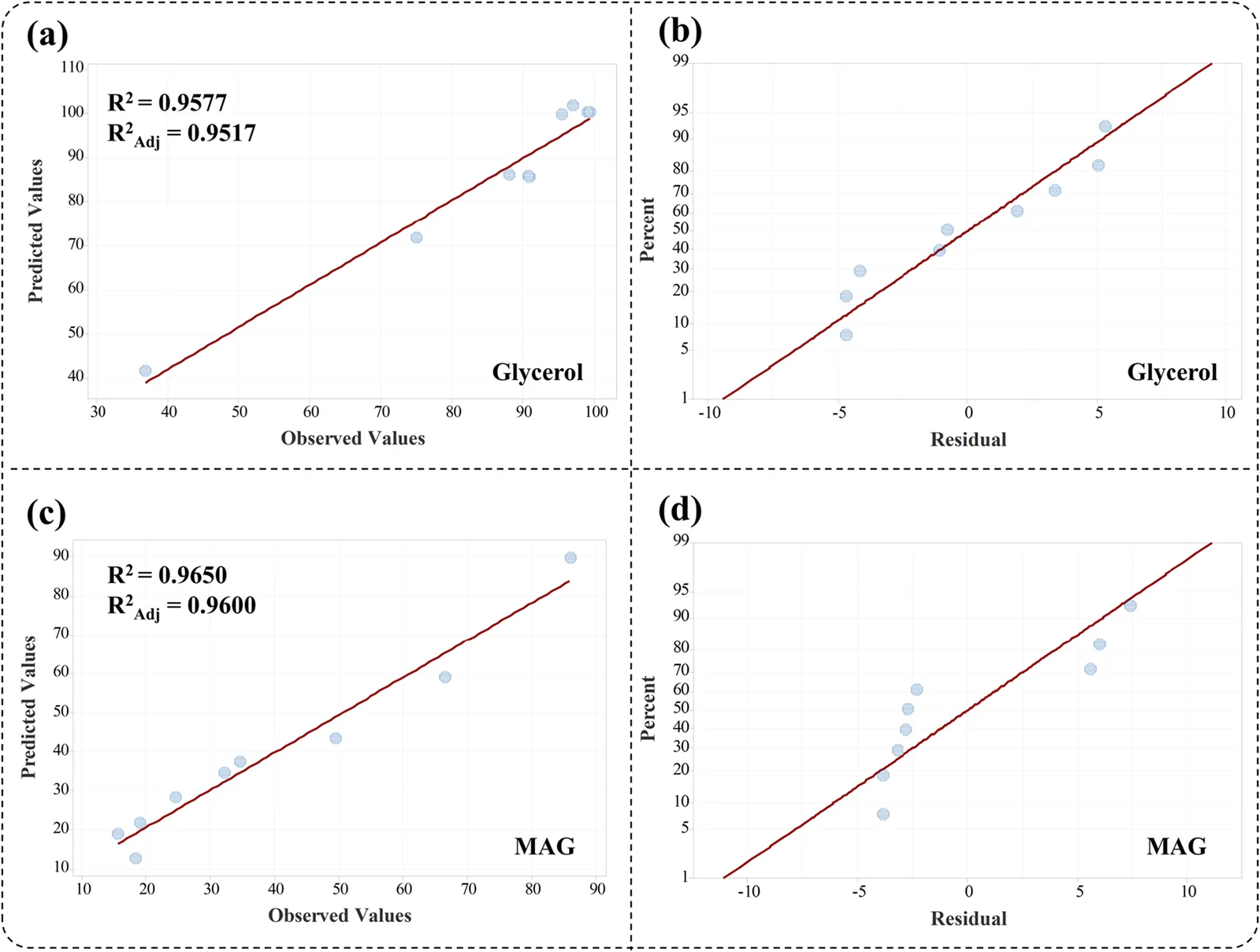
(a) Graph of predicted values versus observed values for glycerol conversion, (b) residual analysis of the statistical model for glycerol conversion, (c) graph of predicted values versus observed values for MAG selectivity, and (d) residual analysis of the statistical model for MAG selectivity.

Figure 10 displays the predicted versus observed value plots and the respective residual analyses for the statistical models of DAG and TAG selectivity. It is observed in Figure 10(a) and 10(c) that the experimental data for both responses are distributed close to their respective regression lines, within the established confidence interval. The high values of the coefficients of determination (*R* ^2^ and adjusted *R* ^2^) of 0.9612, 0.9557, 0.9611, and 0.9555, respectively, corroborate this strong correlation, indicating that the models explain 95.57% and 95.55% of the experimental variability for DAG and TAG formation, respectively. Finally, the residual analyses (Figure 10(b) and 10(d)) show a random distribution of errors around the zero residual line, validating the assumption of normality and the nonsignificance of the residuals for the proposed models.

**Figure 10:**
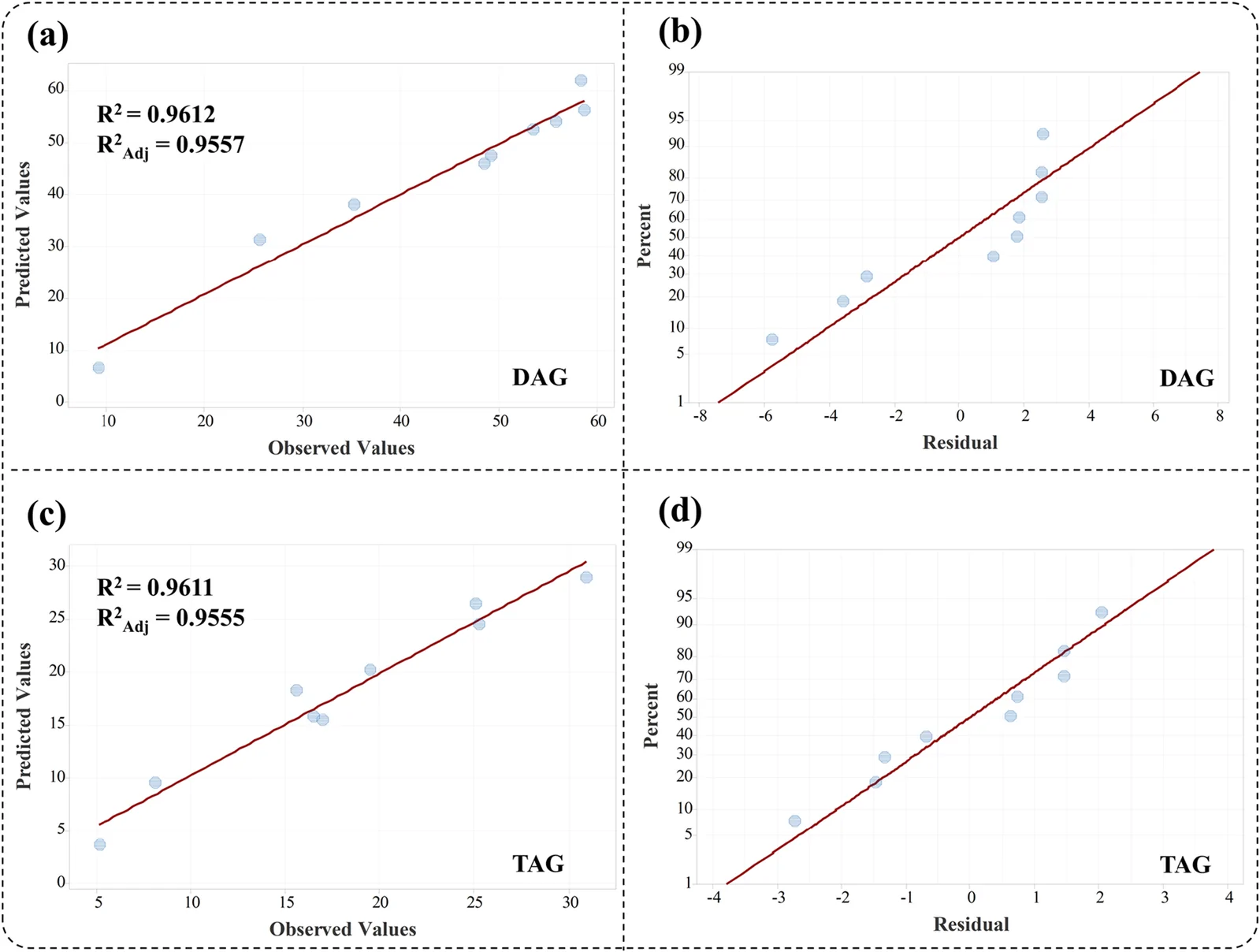
(a) Graph of predicted values versus observed values for DAG selectivity, (b) residual analysis of the statistical model for DAG selectivity, (c) graph of predicted values versus observed values for TAG selectivity, and (d) residual analysis of the statistical model for TAG selectivity.

### 3.3 Effect of Interactions between Independent Variables on Glycerol Conversion and MAG, DAG, and TAG Selectivity

To optimize the independent variables of the process, the effects of their interactions on the response variables, glycerol conversion, and MAG, DAG, and TAG selectivity, were analyzed. The interactions among temperature (A), time (B), catalyst concentration (C), and glycerol:acetic acid molar ratio (D) were evaluated by means of six contour plots, one for each response variable. Each contour plot was generated by varying two independent variables at a time while keeping the others fixed at their central levels (level 2 of the Taguchi orthogonal design).

Figure 11 displays the contour plots for glycerol conversion and MAG selectivity. Analysis of Figure 11(a–f) reveals that the highest glycerol conversion values are achieved at temperatures above 90 °C and glycerol:acetic acid molar ratios exceeding 1:3. This behavior is directly associated with the fact that these variables exhibit the first and second highest percentage contributions, respectively, combined with the endothermic nature of the reaction and the greater availability of acetic acid in the medium, which shifts the equilibrium toward acetal formation.^9,14^ Conversely, increasing reaction time and catalyst concentration also contribute positively to conversion, albeit to a lesser extent. The optimal ranges observed in the contour plots lie above 2 h of reaction and 5% catalyst loading, which is consistent with the third and fourth positions occupied by these variables in the contribution ranking.

**Figure 11:**
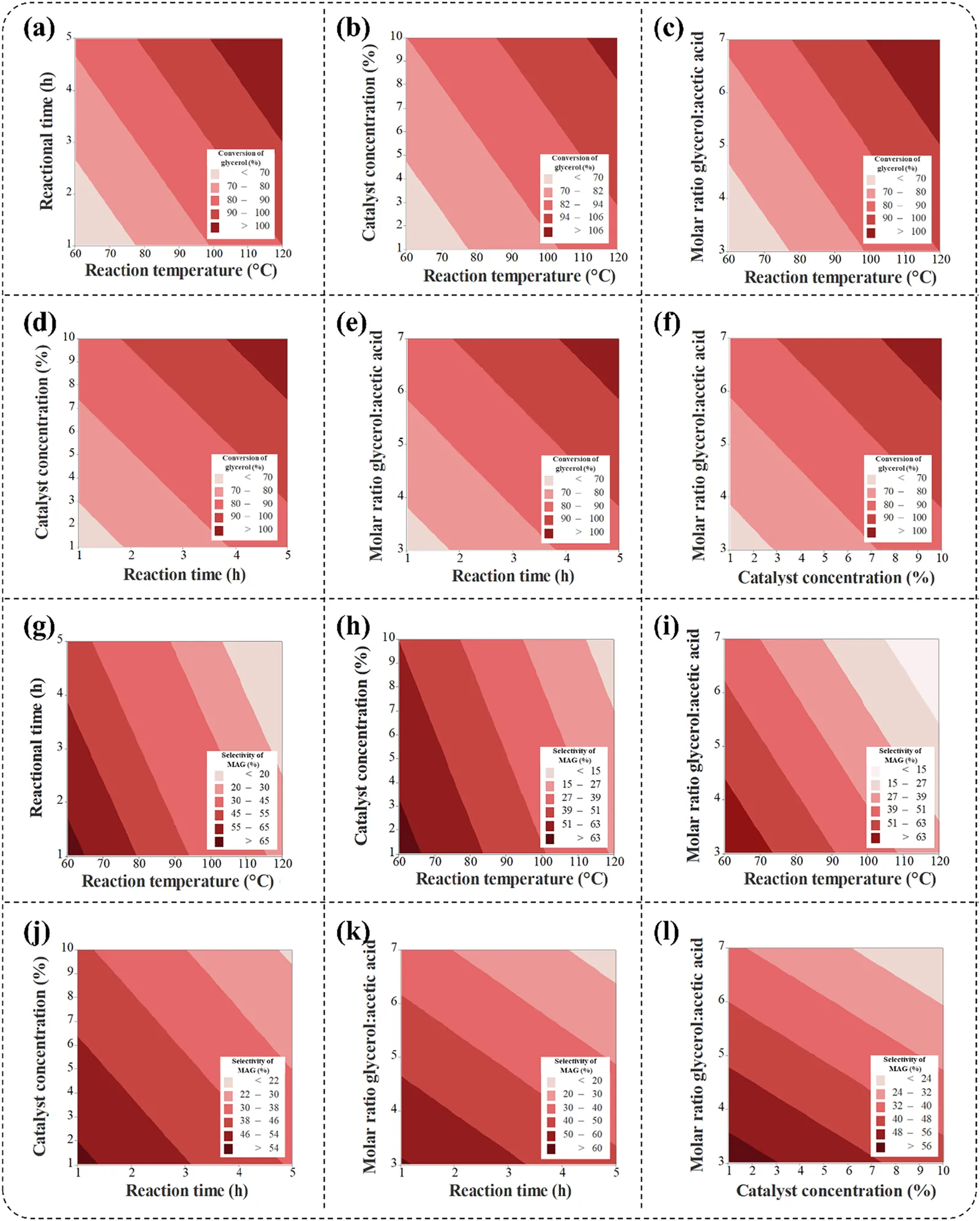
Contour plots for glycerol conversion (%) as a function of: (a) reaction temperature and reaction time, (b) reaction temperature and catalyst concentration, (c) reaction temperature and molar ratio, (d) reaction time and catalyst concentration, (e) reaction time and molar ratio, and (f) catalyst concentration and molar ratio. Contour plots for MAG selectivity as a function of: (g) reaction temperature and reaction time, (h) reaction temperature and catalyst concentration, (i) reaction temperature and molar ratio, (j) reaction time and catalyst concentration, (k) reaction time and molar ratio, and (l) catalyst concentration and molar ratio.

On the other hand, analysis of Figure 11(g–l) reveals that MAG selectivity exhibits an opposite trend to glycerol conversion. The highest MAG selectivities are achieved under milder reaction conditions, particularly at temperatures below 80 °C and glycerol:acetic acid molar ratios below 1:4, situations in which the initial formation of monoacetin is favored, while progression to DAG and TAG is limited. However, increasing temperature and the amount of acetic acid raise the kinetic energy and accelerate the reaction, whereas an excess of acetic acid reduces the time required to reach equilibrium, favoring the conversion of MAG to DAG.^12,15^ Furthermore, it is observed in Figure 11(g–l) that MAG selectivity is favored by shorter reaction times and lower catalyst concentrations, since prolonged reaction times promote the successive substitution of hydroxyl groups by acetate groups, and higher concentrations of active sites intensify the interactions between reactants, leading to the formation of diacetins and triacetins and, consequently, to a reduction in the MAG fraction.^4,20^

Figure 12 presents the contour plots for DAG and TAG selectivity. Analysis of the contour surfaces for DAG selectivity reveals that both are favored under harsher reaction conditions compared to those observed for MAG formation. The highest glycerol conversions to DAG occur at temperatures above 100 °C, reaction times exceeding 2 h, catalyst concentrations above 5%, and glycerol:acetic acid molar ratios above 1:5. These conditions promote the conversion of monoacetin to diacetin due to the endothermic nature of the reaction, the increase in kinetic energy and higher frequency of effective collisions between reactants, as well as the enhanced interaction with active sites provided by prolonged reaction times.^2,18,65^

**Figure 12:**
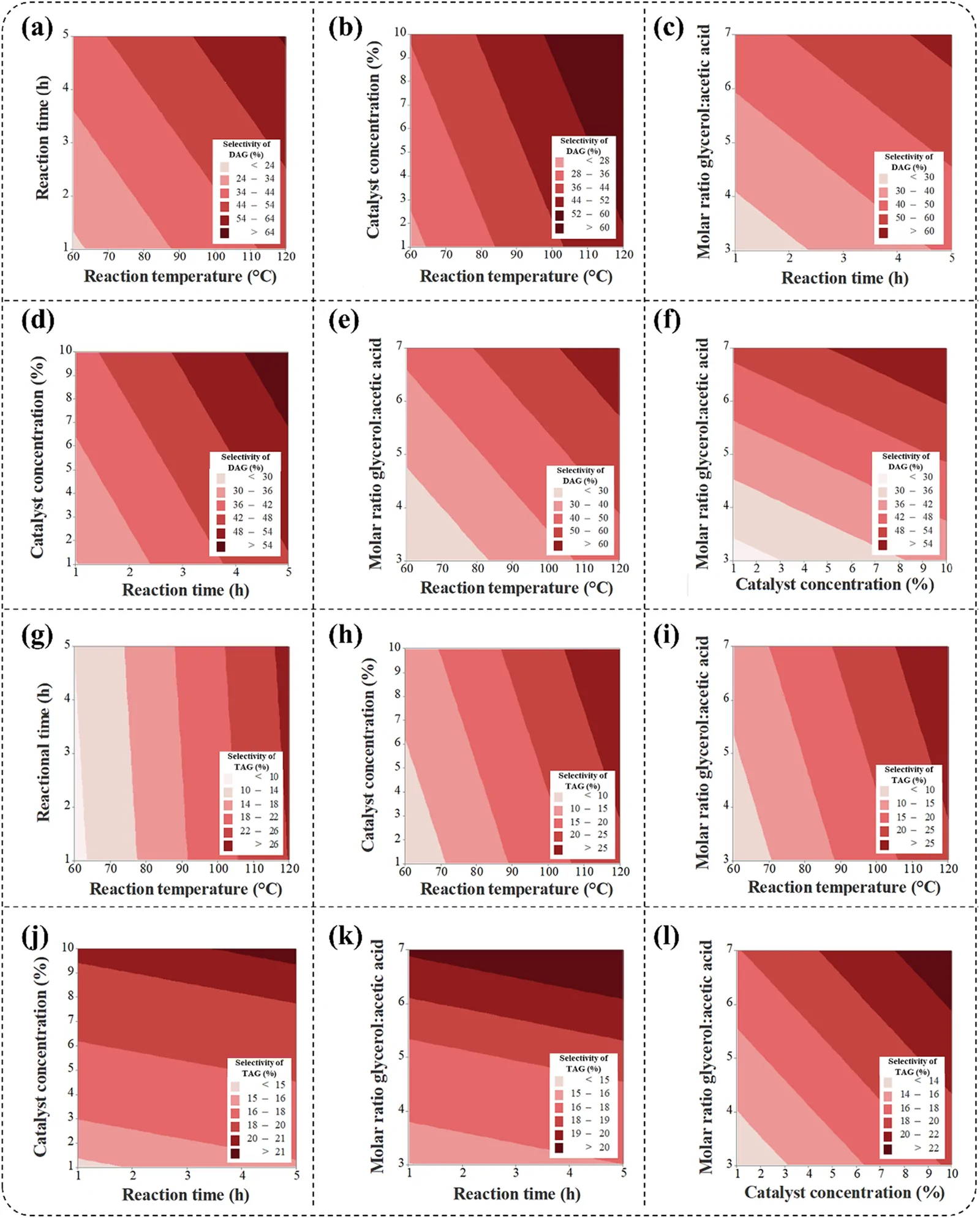
Contour plots for DAG selectivity (%) as a function of: (a) reaction temperature and reaction time, (b) reaction temperature and catalyst concentration, (c) reaction temperature and molar ratio, (d) reaction time and catalyst concentration, (e) reaction time and molar ratio, and (f) catalyst concentration and molar ratio. Contour plots for TAG selectivity as a function of: (g) reaction temperature and reaction time, (h) reaction temperature and catalyst concentration, (i) reaction temperature and molar ratio, (j) reaction time and catalyst concentration, (k) reaction time and molar ratio, and (l) catalyst concentration and molar ratio.

TAG selectivity exhibits this same trend toward reaction conditions in an even more pronounced manner, being maximized when the temperature exceeds 100 °C, the reaction time surpasses 2 h, the catalyst concentration exceeds 7%, and the molar ratio remains above 1:5. Under these conditions, a high formation of triacetin occurs due to sufficient energy availability, adequate time for reactant interaction, and the presence of active sites that promote the complete substitution of glycerol hydroxyl groups.^9,30^ However, it should be noted that although increasing temperature and catalyst concentration intensify interactions and accelerate the reaction, elevated temperatures may lead to complete volatilization of acetic acid, while excessive catalyst concentrations can increase the medium viscosity and impair mass transfer.^14,19^ Conversely, excessively high molar ratios may dilute the system, reducing the efficiency of contact between glycerol and acetic acid and, consequently, the extent of subsequent reactions.^66^

Thus, the results suggest the existence of an optimal range for each independent variable, within which samples with high glycerol conversion and high DAG and TAG selectivities are obtained. Accordingly, when performing the acetylation reaction using the CMS750-NiFe_2_O_4_ catalyst under reaction conditions in the ranges of 90–105 °C (reaction temperature), 2–3 h (reaction time), 1:4–1:6 (glycerol:acetic acid molar ratio), and 5–8% (catalyst concentration), products with high glycerol conversion and high DAG and TAG selectivity are achieved.^66^

### 3.4 Optimization of Reaction Variables and Statistical Model Validation

The predictive capability of the statistical models developed in this study was evaluated for different glycerol conversion and MAG, DAG, and TAG selectivity values by performing three additional experiments. Experiments 1 and 2 were conducted under random reaction conditions, while Experiment 3 consisted of a repetition of the conditions used in Experiment 2, aiming to verify the reproducibility of the results. Table 11 presents the glycerol conversion and MAG, DAG, and TAG selectivity values predicted by the models, as well as the experimentally obtained values.

**Table 11:** Results of the Validation of Predictive Models Developed for Glycerol Conversion and Selectivity for MAG, DAG, and TAG^a^,^b^

	Reaction Conditions				Glycerol Conversion (%)			Selectivity of MAG (%)			Selectivity of DAG (%)			Selectivity of TAG (%)		
Experiment	*A*	*B*	*C*	*D*	PV	OV	RE	PV	OV	RE	PV	OV	RE	PV	OV	RE
**1**	60	0.5	1:3	1.5	40.33	42.20	4.63	91.14	90.85	0.31	4.92	5.13	4.26	3.83	4.02	4.96
**2**	80	1.5	1:5	4.0	70.25	72.66	3.43	54.97	52.85	3.85	31.31	32.53	3.89	13.93	14.62	4.95
**3**	80	1.5	1:5	4.0	70.25	72.86	3.71	54.97	52.98	3.62	31.31	32.73	4.53	13.93	14.35	3.01

a *A*, *B*, *C*, and *D* denote reaction temperature, reaction time, catalyst loading, and glycerol:acetic acid molar ratio, respectively.

b PV: predicted value; observed value; and RE: relative errors.

Analysis of the results presented in Table 11 shows that the experiments carried out under random reaction conditions resulted in samples with glycerol conversions of 42.20% (Experiment 1) and 72.66% (Experiment 2), with relative errors of 4.63% and 3.43%, respectively. Regarding selectivity, experiment 1 exhibited values of 90.85% for MAG, 5.13% for DAG, and 4.02% for TAG, with relative errors of 0.31%, 4.26%, and 4.96%, respectively. Experiment 2, in turn, resulted in selectivities of 52.85% (MAG), 32.53% (DAG), and 14.62% (TAG) with relative errors of 3.85%, 3.89%, and 4.95%. Experiment 3, which consisted of a repetition of the conditions used in experiment 2, achieved a glycerol conversion of 72.86% (relative error of 3.71%) and selectivities of 52.98% (MAG), 32.73% (DAG), and 14.35% (TAG), with relative errors of 3.62%, 4.53%, and 3.01%, respectively. These results demonstrate that the predictive models developed in this study are valid, exhibit high reliability, and are effective in predicting experimental outcomes and optimizing reaction variables within the established 95% confidence interval.^23^

### 3.5 Catalyst Reusability

The application of heterogeneous acid catalysts with magnetic properties in the glycerol acetylation reaction is advantageous, as these solids can be magnetically separated and reused in successive cycles, eliminating the need for conventional separation steps. Thus, after optimizing the reaction parameters for acetylation, the CMS750-NiFe_2_O_4_ catalyst was subjected to successive reuse cycles to evaluate its catalytic activity and selectivity in acetal formation. Figure 13 presents the catalyst reuse study under the optimized reaction conditions: temperature of 120 °C, reaction time of 2.5 h, catalyst concentration of 7.5%, and glycerol:acetic acid molar ratio of 1:6.

**Figure 13:**
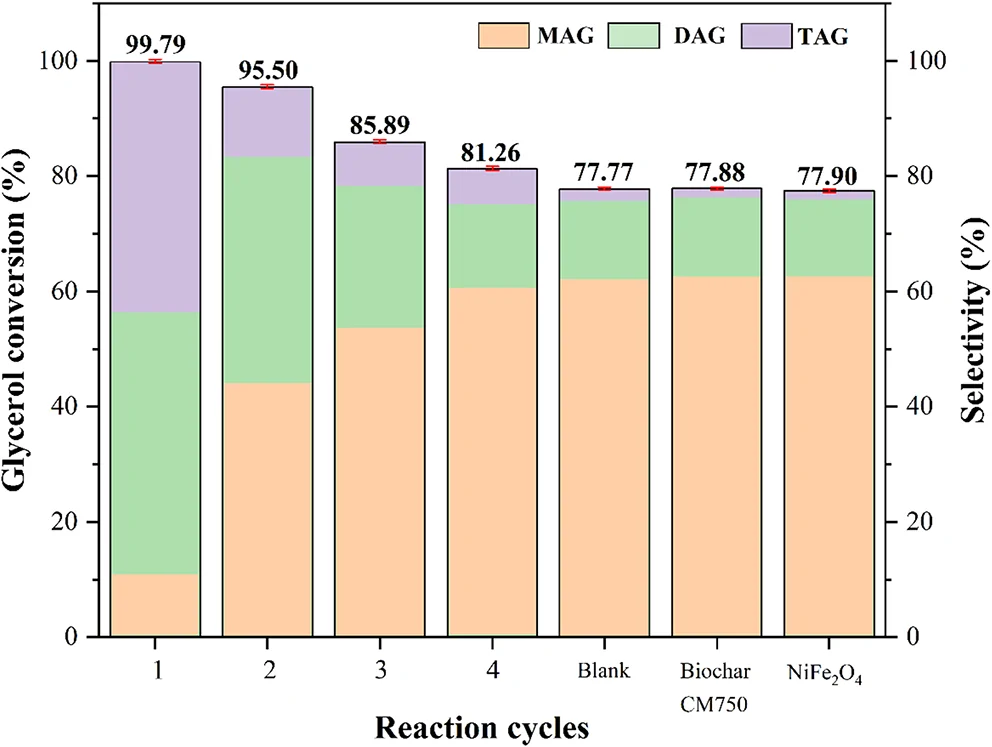
Glycerol conversion and selectivity for MAG, DAG, and TAG throughout the reuse cycles of the CMS750–NiFe_2_O_4_ catalyst in the glycerol acetylation reaction.

The results presented in Figure 13 show that, in the first reaction cycle, the CMS750-NiFe_2_O_4_ catalyst provides high glycerol conversion (99.79% ± 0.55), along with high selectivity for the more substituted products, DAG (40.16%) and TAG (50.16%), and low selectivity for MAG (9.58%), as illustrated in the chromatogram of the sample obtained in the first cycle (Figure 14(a) and (b)). These findings indicate that the catalyst possesses acidic sites capable of promoting the sequential conversion of glycerol into the more substituted acetals. However, over subsequent reuse cycles, although glycerol conversion remains high, 95.50% ± 0.45, 85.89% ± 0.40, and 81.26% ± 0.44 in the second, third, and fourth cycles, respectively, a significant decrease is observed in the formation of the more substituted acetals (DAG and TAG), accompanied by an increase in MAG formation (from 9.58% in the first cycle to 65.44% in the fourth cycle). This behavior indicates that the catalyst remains catalytically active after four reuse cycles; however, it undergoes partial deactivation, which is mainly reflected in the loss of selectivity toward the more substituted products (DAG and TAG).^2,19^

**Figure 14:**
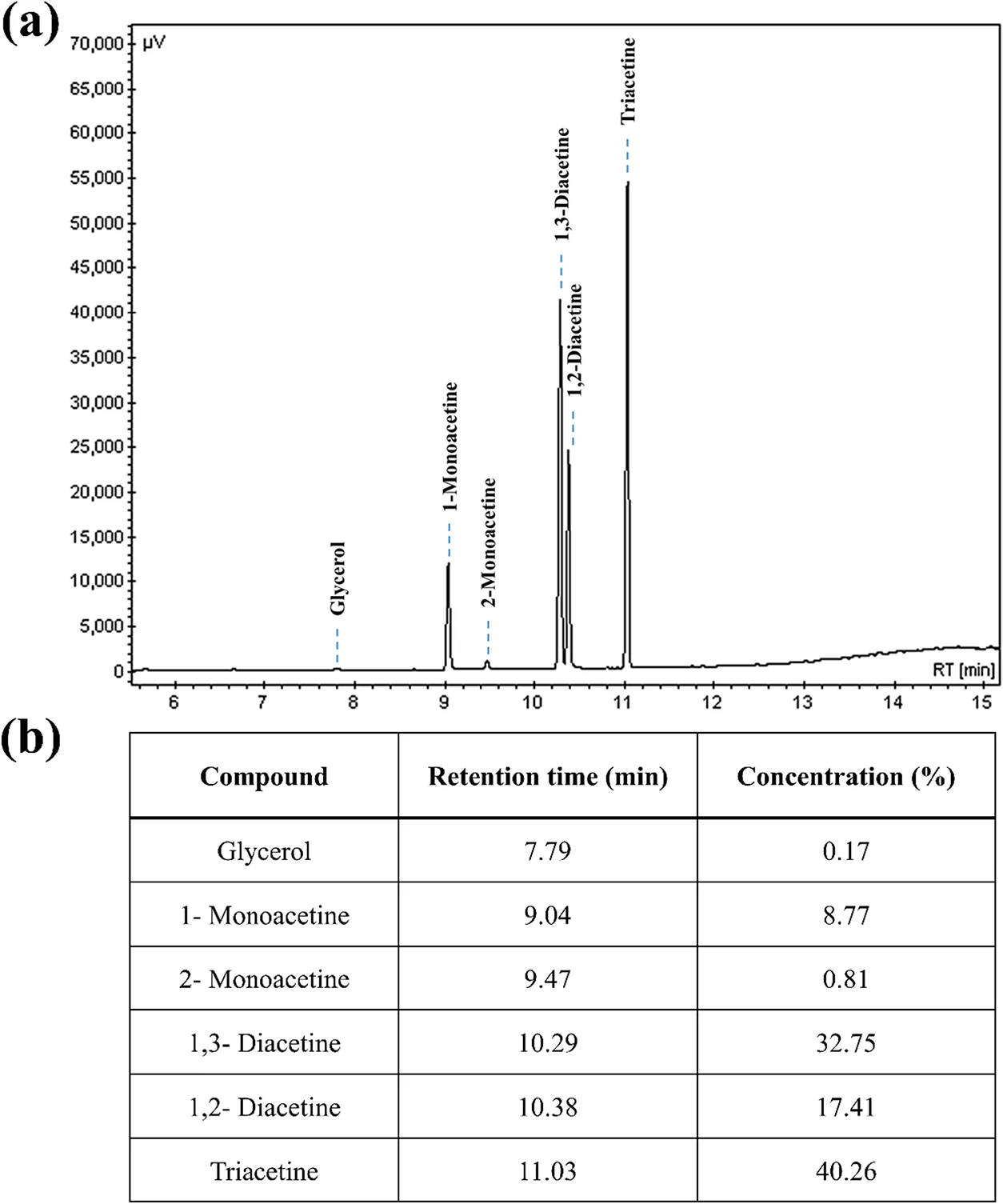
(a) GC chromatogram and (b) selectivity of acetylation products obtained from glycerol acetylation using the CMS750–NiFe_2_O_4_ catalyst.

In order to verify whether the impairment of the catalyst’s selective action was caused by the leaching of sulfonic groups, EDS and surface acidity analyses were performed on the catalyst recovered after the 4th reaction cycle. The results are presented in Figure S2 and Table S1. The results obtained for surface elemental composition (EDS) show a decrease in sulfur content from 4.54% ± 0.185 (fresh catalyst) to 2.47% ± 0.165 (catalyst after the 4th cycle). Furthermore, the same downward trend was observed in the surface acidity values determined for the fresh catalyst and after the 4th cycle, which were 1.36 ± 0.028 and 0.75 ± 0.035 mmol H^+^ g^–1^, respectively. These results suggest that the impairment of catalyst selectivity is associated with partial leaching of sulfonic groups over the reaction cycles.

It is worth noting that, despite the decrease in catalytic efficiency of the CMS750-NiFe_2_O_4_ catalyst over successive reaction cycles, it still exhibits, in the fourth cycle, glycerol conversion and selectivities to MAG, DAG, and TAG considerably higher than those obtained in the control experiments (CM750 biochar only, NiFe_2_O_4_ only, and the blank reaction, without catalyst). The reaction using only the biochar showed a glycerol conversion of 77.88%±0.20 and selectivities of 68.69% (MAG), 29.92% (DAG), and 1.39% (TAG). In turn, the reaction in the presence of NiFe_2_O_4_ resulted in a conversion of 77.90% and selectivities of 68.65% (MAG), 29.98% (DAG), and 1.40% (TAG). Similarly, the blank reaction exhibited a conversion of 77.77% ± 0.33, with selectivities of 68.63% (MAG), 29.95% (DAG), and 1.43% (TAG). These findings reinforce that the observed catalytic activity is predominantly attributed to the presence of sulfonic groups on the surface of the CMS750-NiFe_2_O_4_ catalyst.

This same behavior is widely reported in the literature for heterogeneous acid catalysts employed in the glycerol acetylation reaction. Termezi et al.,^2^ evaluating Fe–Ni/AC catalysts, observed that in the blank test conducted at 90 °C, with a reaction time of 1 h and a glycerol:acetic acid molar ratio of 1:3, the glycerol conversion was 68.0%, with selectivities of 35.23% for MAG and 64.77% for DAG. The authors attributed these values to the presence of acetic acid in the reaction medium, which exerts an autocatalytic effect, since the reaction is acid-catalyzed. Furthermore, it was found that introducing the best-performing catalyst (5Fe–Ni/AC) into the reaction system increased glycerol conversion to 88.8%, accompanied by selectivities of 55.86% for MAG, 42.31% for DAG, and 0.87% for TAG.

Similarly, Fronza et al.^6^ reported glycerol conversions above 80%, with selectivities of 40.0% for MAG, 49.0% for DAG, and 11.0% for TAG, also attributing these results to the autocatalytic effect of acetic acid on the reaction medium. The authors further observed that introducing the best-performing catalyst, NbOPO_4_, increased glycerol conversion to values above 99.8%, with selectivities of 29.07% for MAG, 55.30% for DAG, and 15.63% for TAG. Thus, these findings corroborate that the maintenance of selectivity toward the more substituted acetals, DAG, and TAG, is strongly associated with the stability and strength of the acid sites of the CMS750-NiFe_2_O_4_ catalyst.

Finally, a decrease in the magnetic properties of the catalyst recovered after the fourth reaction cycle was also observed. The surface elemental composition results (EDS), presented in Figure S2a, show a reduction in iron content from 2.64% ± 0.115 (fresh catalyst) to 2.22% ± 0.113 (catalyst after the fourth cycle), as well as a decrease in nickel content from 1.54% ± 0.125 to 1.30% ± 0.105%. It is worth noting that, despite this reduction, the Fe/Ni ratio remained close to the theoretical stoichiometric ratio of 2:1 for the NiFe_2_O_4_ phase. Furthermore, the same trend was observed in the VSM analysis (Figure S2b), which showed a decrease in saturation magnetization from 4.11 emu g^–1^ (fresh catalyst) to 2.91 emu g^–1^ (catalyst recovered after the fourth cycle). The decrease in the surface contents of iron and nickel, constituents of the NiFe_2_O_4_ magnetic phase, can be attributed to the partial leaching of these metals over the successive catalyst reuse cycles. However, this reduction does not significantly compromise the separation of the catalyst from the reaction medium by applying an external magnetic field, as evidenced in Figure S2b.

### 3.6 Comparison of the CMS750-NiFe_2_O_4_ Catalyst with Different Catalysts

The catalytic performance of CMS750-NiFe_2_O_4_ in the glycerol acetylation reaction was compared with that of other acid catalysts reported in the literature, as shown in Table S2. The results show that the catalyst developed in this study achieves high glycerol conversion (99.79%) within only 2.5 h of reaction time, which is shorter than those reported for sulfonated peanut shell carbon (APC–F), sulfonated carbon derived from glucose (Gluc_1:2), and the BuSnCl_3_ catalyst, which require reaction times longer than 3 h to reach conversions of 99.0%, 100.0%, and 100%, respectively.^7,19,14^ In addition to the high conversion in a short reaction time, CMS750-NiFe_2_O_4_ exhibited higher selectivity toward triacetin (TAG), the most valuable product, reaching 50.16%, while APC–F, Gluc_1:2, and BuSnCl_3_ showed selectivities of 16.10%, 33.0%, and 30.8%, respectively. Conversely, the DAG selectivities were 40.16% (CMS750-NiFe_2_O_4_), 50.10% (APC–F), 56.0% (Gluc_1:2), and 43.5% (BuSnCl_3_). These results demonstrate that the catalyst developed in this study favors the formation of the most substituted product in shorter reaction times, reducing energy consumption and making CMS750-NiFe_2_O_4_ a more efficient alternative from an operational standpoint.

Furthermore, the catalytic tests performed in this study employed a catalyst concentration lower than that reported for the APC–F, TRG–BDS, ACmicro–BDS, PC–C, Gluc_1:2, BuSnCl_3_, and 30NiO/TiO_2_ systems, which require concentrations equal to or higher than 9.0% to achieve glycerol conversions above 88.0%, with DAG and TAG selectivities comparable to those obtained with CMS750-NiFe_2_O_4_.^7,18,8,16,19,13,14^ These results evidence the high catalytic efficiency of the developed material.

It is also observed that some of the catalysts described in the literature were not subjected to reuse studies, such as ACmicro–BDS, PC–C, 5Fe–5Ni/AC, and BuSnCl_3_.^8,16,2,14^ Among the materials evaluated in Table S2, CMS750-NiFe_2_O_4_ stood out for its good performance, maintaining glycerol conversion above 81.0% after four reaction cycles, a value higher than the initial conversion reported for APC–F, TRG–BDS, and 30NiO/TiO_2_, with a number of reuse cycles similar to that described for these systems.^7,18,13^

Although the 30NiO/TiO_2_ catalyst exhibits a higher number of cycles, its application requires more severe conditions, including a temperature of 170 °C and a catalyst concentration of 29.0%, to achieve a glycerol conversion of 90.2%.^13^ These results demonstrate that CMS750-NiFe_2_O_4_ combines high catalytic activity with milder operating conditions compared to several systems reported in the literature. Finally, although a gradual reduction in selectivity toward the more acetylated products is observed over the cycles, the catalyst performance remains competitive when compared to some reported catalysts, whose conversions and selectivities, even in the first cycle, are lower than those achieved by CMS750-NiFe_2_O_4_ after four reuses, as observed for the 5Fe–5Ni/AC catalyst.^19^

Thus, the CMS750-NiFe_2_O_4_ catalyst exhibits high potential for application in the glycerol acetylation reaction, combining high conversion, high selectivity for triacetin under optimized conditions, and maintained catalytic activity over reuse cycles, features that make it a promising alternative for the catalytic valorization of glycerol.

## 4 Conclusion

The present work proposed the synthesis of a novel magnetic sulfonated acid catalyst, obtained from an Amazonian agro-industrial residue, for the catalytic conversion of glycerol into higher value-added products. The characterizations performed, including SA, TGA, XRD, FT-IR, SEM, EDS, and VSM, confirmed the success of the sulfonation and magnetic phase (NiFe_2_O_4_) impregnation processes onto the CM750 biochar structure. Optimization of the reaction variables for the acetylation of glycerol with acetic acid was carried out using Taguchi orthogonal experimental design. The CMS750-NiFe_2_O_4_ catalyst, when applied under the optimized conditions of temperature of 120 °C, reaction time of 2.5 h, catalyst concentration of 7.5%, and glycerol:acetic acid molar ratio of 1:5, achieved a maximum glycerol conversion of 99.79%, with high selectivity toward the more substituted acetals, DAG (40.16%) and TAG (50.16%), and low selectivity for MAG (9.58%). The predictive models developed for glycerol conversion and for MAG, DAG, and TAG selectivities exhibited high goodness of fit (adjusted *R* ^2^ > 0.95) and relative errors below 5%. The CMS750-NiFe_2_O_4_ catalyst maintained catalytic activity after four reuse cycles, with glycerol conversion above 81% and selectivities of 65.44% (MAG), 35.82% (DAG), and 1.75% (TAG). Thus, this study demonstrates the feasibility of using the murici seed agro-industrial residue in the synthesis of magnetic acid catalysts, as well as highlights the possibility of reintegrating residual or low-cost raw materials into productive processes, such as acetal production.

## Supplementary Material



## Notes

EDS spectrum and surface elemental mapping of NiFe_2_O_4_ (Figure S1); EDS and VSM analyses of fresh and spent CMS750-NiFe_2_O_4_ catalysts (Figure S2); surface acidity of the fresh catalyst and after the 4th reaction cycle (Table S1); performance comparison between the CMS750-NiFe_2_O_4_ catalyst and literature solid acid catalysts (Table S2) (PDF)
