# Supplementary material for: Efficient and Reusable Magnetic Sulfonated Biochar from Amazonian Waste for Sustainable Production of Glycerol Acetals: Statistical Optimization of Catalytic Process
Source: ACS Omega. 2026 Sep 17;11(38):57560–82. doi: 10.1021/acsomega.6c06154 (PMC13629393; doi:10.1021/acsomega.6c06154)
Supplement: Supplementary file 1 [file ao6c06154_si_001.pdf]

Supplementary material

Efficient and Reusable Magnetic Sulfonated Biochar from Amazonian Waste for Sustainable Production of Glycerol Acetals: Statistical Optimization of Catalytic Process

Thaissa Saraiva Ribeiro[a], Izadora de Araújo Sobrinho[a], Ane Caroline Dias e Silva[a], Beatriz dos Santos Silva[a], Deborah da Cunha Fonseca[b], Vicente da Silva Lima[c], Matheus Arrais Gonçalves[a], Alcineia Conceição Oliveira[d,e], Igor Alexandre Rocha Barreto[f], Marcondes Lima da Costa[f], Leyvison Rafael Vieira da Conceição[a,b*].

[a] Federal University of Pará, Institute of Exact and Natural Sciences, Graduate in Chemistry Program, Laboratory of Catalysis and Oleochemical, 66075–110, Belém, Pará, Brazil.

[b] Federal Institute of Education, Science and Technology of Pará, Graduate in Materials Engineering Program, 66093–020 Belém, Pará, Brazil.

[c] Federal University of Pará, Institute of Biological Sciences, Graduate Program in Biotechnology, Amazon Oils Laboratory, 66075–110, Belém, Pará, Brazil.

[d] Federal University of Ceará, Science Center, Department of Analytical Chemistry and Physical Chemistry, Graduate Program in Chemistry, Physical Chemistry Laboratory, 60440-900, Fortaleza, Ceará, Brazil.

[e] Federal University of Ceará, Science Center, Department of Analytical Chemistry and Physical Chemistry, Graduate Program in Chemical Engineering, Physical Chemistry Laboratory, 60440-900, Fortaleza, Ceará, Brazil.

[f] Federal University of Pará, Program for Post-Graduation in Geology and Geochemistry, Institute of Geosciences, 66075-110, Belém, Pará, Brazil.

*Corresponding author

E-mail address: rafaelvieira@ufpa.br (Conceição, L.R.V)

Figure S1. (a) EDS spectrum and (b) surface elemental mapping of $NiFe_2O_4$.

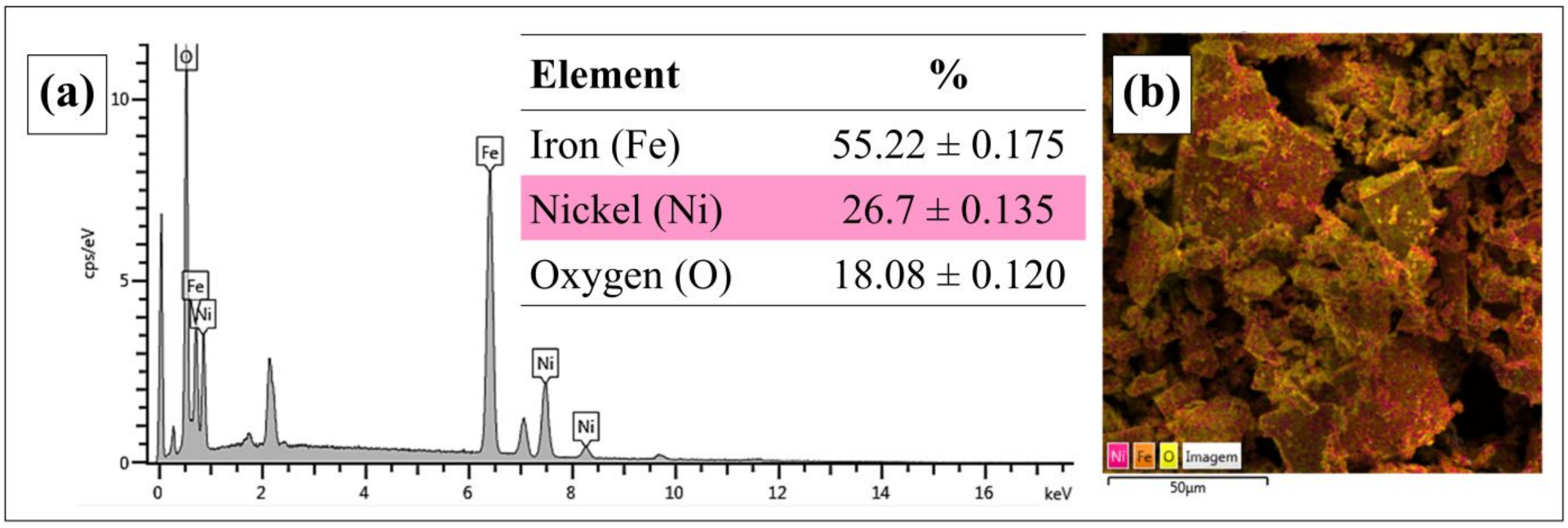

Element	%
Iron (Fe)	55.22 ± 0.175
Nickel (Ni)	26.7 ± 0.135
Oxygen (O)	18.08 ± 0.120

Figure S2. Analysis of (a) EDS and (b) VSM of CMS750- $NiFe_2O_4$ catalysts and catalyst recovered after the 4th cycle.

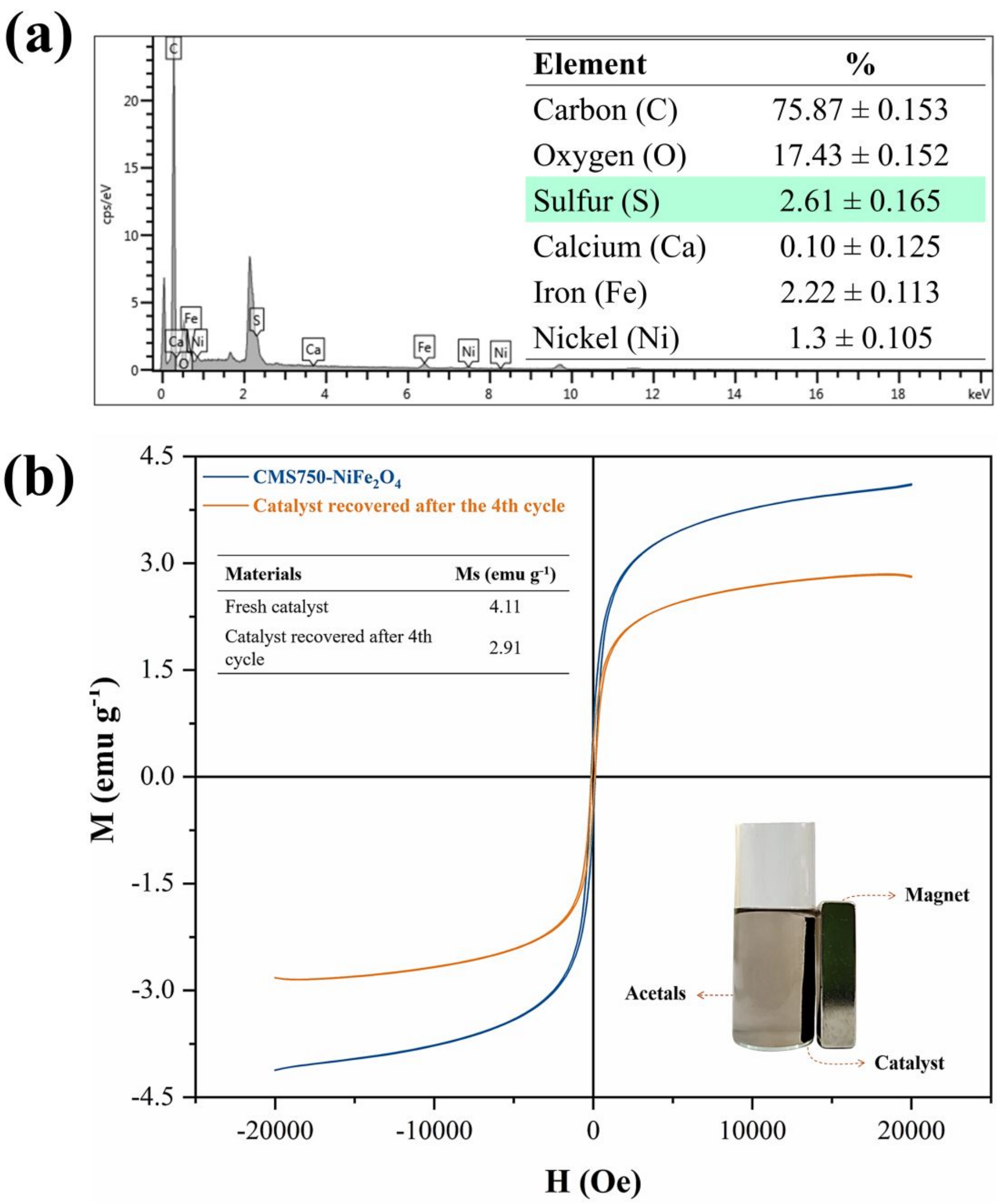

Element	%
Carbon (C)	75.87 ± 0.153
Oxygen (O)	17.43 ± 0.152
Sulfur (S)	2.61 ± 0.165
Calcium (Ca)	0.10 ± 0.125
Iron (Fe)	2.22 ± 0.113
Nickel (Ni)	1.3 ± 0.105

Materials	Ms (emu g^{-1})
Fresh catalyst	4.11
Catalyst recovered after 4th cycle	2.91

Table S1. Analysis of the surface acidity of the catalysts before the reaction and recovered after the 4th reaction cycle.

**Sample**	**Surface acidity (mmol H^+ g^{-1})**
Catalyst before 1° reactional cycle	1.368 ± 0.028
Catalyst after 4° reactional cycle	0.759 ± 0.015

Table S2. Performance comparison between the solid acid catalysts reported in the literature and the CMS750-$NiFe_2O_4$ catalyst developed in this study.

**Catalyst**	**Reaction conditions**				**Glycerol conversion (%)**	**Selectivity (%)**				**Reaction cycles**	**Reference**
	T (°C)	t (h)	RM	Catalyst conc. (%)		MAG	DAG	TAG	DAG+TAG		
5Fe–5Ni/AC	90	1.0	1:3	3.0	88.0	56.82	42.31	0.87	43.18	–	[2]
APC–F	120	4.0	1:6	25.0	99.0	29.40	54.10	16.10	70.20	5	[7]
ACmicro–BDS	110	1.0	1:6	9.0	100.0	8.0	50.0	42.0	92.0	–	[8]
30NiO/TiO_2	170	0.5	1:10	29.0	90.2	19.6	14.5	65.9	80.4	8	[13]
$BuSnCl_3$	80	3.0	1:4	–	100.0	25.7	43.5	30.8	74.3	–	[14]
PC–C	110	2.0	1:6	9.0	97.0	24.0	55.0	21.0	76.0	–	[16]
TRG–BDS	110	1.0	1:6	9.0	95.0	27.00	–	–	63.0	4	[18]
Gluc_1:2	120	5.0	1:7.8	10.0	100.0	12.0	56.0	33.0	89.0	3	[19]
CMS750-$NiFe_2O_4$	120	2.5	1:5	7.5	99.79	9.58	40.16	50.16	90.32	4	**This study**
